# Glass Transition Temperatures of Organic Mixtures from Isoprene Epoxydiol-Derived Secondary Organic Aerosol

**DOI:** 10.1021/acs.jpca.2c08936

**Published:** 2023-05-02

**Authors:** Bo Chen, Jessica A. Mirrielees, Yuzhi Chen, Timothy B. Onasch, Zhenfa Zhang, Avram Gold, Jason D. Surratt, Yue Zhang, Sarah D. Brooks

**Affiliations:** † Department of Atmospheric Sciences, Texas A&M University, Eller O&M Building, 1204, 3150 TAMU, 797 Lamar Street, College Station, Texas 77843, United States; ‡ Department of Chemistry, University of Michigan, 930 N University Avenue, Ann Arbor, Michigan 48104, United States; § Gillings School of Global Public Health, Department of Environmental Sciences and Engineering, University of North Carolina at Chapel Hill, 170 Rosenau Hall, Campus Box #7400, 135 Dauer Drive, Chapel Hill, North Carolina 27599, United States; ∥ Aerodyne Research, Inc, 45 Manning Road, Billerica, Massachusetts 01821, United States; ⊥ College of Arts and Sciences, Department of Chemistry, University of North Carolina at Chapel Hill, Campus Box #3290, 125 South Road, Chapel Hill, North Carolina 27599, United States

## Abstract

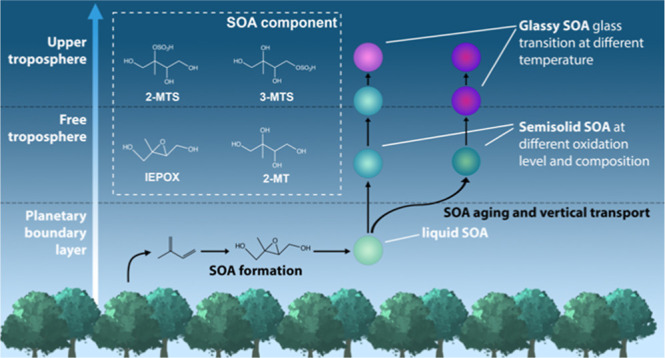

The phase states and glass transition temperatures (*T*_g_) of secondary organic aerosol (SOA) particles are important to resolve for understanding the formation, growth, and fate of SOA as well as their cloud formation properties. Currently, there is a limited understanding of how *T*_g_ changes with the composition of organic and inorganic components of atmospheric aerosol. Using broadband dielectric spectroscopy, we measured the *T*_g_ of organic mixtures containing isoprene epoxydiol (IEPOX)-derived SOA components, including 2-methyltetrols (2-MT), 2-methyltetrol-sulfate (2-MTS), and 3-methyltetrol-sulfate (3-MTS). The results demonstrate that the *T*_g_ of mixtures depends on their composition. The Kwei equation, a modified Gordon–Taylor equation with an added quadratic term and a fitting parameter representing strong intermolecular interactions, provides a good fit for the *T*_g_-composition relationship of complex mixtures. By combining Raman spectroscopy with geometry optimization simulations obtained using density functional theory, we demonstrate that the non-linear deviation of *T*_g_ as a function of composition may be caused by changes in the extent of hydrogen bonding in the mixture.

## Introduction

Secondary organic aerosol (SOA), primarily formed by condensation and reactive uptake of oxidation products of volatile organic compounds (VOCs), accounts for a significant portion of the submicron-sized aerosol in the atmosphere.^1−4^ The conventional assumption was that atmospheric SOA exists in a well-mixed liquid state.^5−7^ Recent studies have shown that lab-generated and atmospheric SOA could become highly viscous and form amorphous solids at atmospherically relevant temperatures and relative humidity (RH).^8−22^ Global modeling predicts that SOA exists mostly in an amorphous state in the middle and upper troposphere.^23^

The particle phase state strongly affects the growth and the atmospheric impact of SOA.^10,24^ Diffusion and reactivity could be significantly limited in highly viscous and glassy SOA.^13,24−30^ It has also been shown that highly viscous and glassy SOA could kinetically limit evaporation and uptake rates; therefore, viscous SOA may not reach equilibrium within an atmospherically relevant time scale.^13,30−39^ Viscous and glassy SOA present in the free troposphere could have a significant impact on ice cloud formation and lifetime, affecting Earth’s radiative balance and climate.^40,41^ At lower temperatures, the slow diffusion rate of water in highly viscous or glassy SOA allows a semi-solid or solid form to persist in contact with water.^42,43^ As a result, glassy SOA can participate in heterogeneous ice nucleation through deposition ice nucleation and immersion freezing.^44−51^

One metric of the particle phase state is the glass transition temperature (*T*_g_). *T*_g_ of pure SOA components and SOA surrogates have been measured using differential scanning calorimetry (DSC),^14^ broadband dielectric spectroscopy (BDS),^19,20^ and the poke-flow technique.^21^ Since SOA often exists as multi-component mixtures comprising organic oxidation products, inorganic components, and water, predicting the mixture *T*_g_ as a function of its composition is particularly important for the modeling of the SOA phase state. Several equations have been developed to predict the *T*_g_ of binary mixtures. One widely used equation is the Fox equation^52^
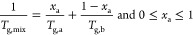
1where *T*_g,a_ and *T*_g,b_ are the glass transition temperature of pure components a and b. In this paper, a is the component that has a lower *T*_g_. *x*_a_ is the mass fraction of component a, and 1 – *x*_a_ is the mass fraction of component b.^53^ The Fox equation is a special case of the Gordon–Taylor equation^53^
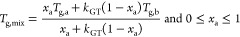
2where *k*_GT_ is the Gordan–Taylor constant, and it can be empirically derived by fitting the Gordon–Taylor equation to measured mixture *T*_g_ and composition relations. It has been proposed that the Fox and the Gordon–Taylor equations can be used to predict the mixture effect on *T*_g_ of SOA.^10^ To study the effect of water content on the *T*_g_ of SOA, the *T*_g_ of butanediol and water mixture have been measured. The results show that the *T*_g_ of the mixture follows the Gordon–Taylor equation.^20^ Binary mixtures comprised sodium nitrate or ammonium bisulfate mixed with one of the various organic compounds were studied, and the *T*_g_ follows the Gordon–Taylor equation when the mixtures are homogeneous.^15^ In addition, measurements show that the *T*_g_ of binary mixtures of two α-pinene oxidation products, 3-methylbutane-1,2,3-tricarboxylic acid, and pinonic acid, not only follows the Gordon–Taylor equation but also shows a linear relation with the *k*_GT_ ≈ 1.^14^ However, the Gordon–Taylor equation can fail when there exists a particularly strong intermolecular interaction between the two pure compounds.^10,20^ For example, measurements show that the *T*_g_ of mixtures of two SOA surrogates, glycerol and 1,2,6-hexanetriol, deviates significantly from the predictions of the Gordon–Taylor equation.^20^ Since the Gordon–Taylor equation is not always accurate, the Kwei equation was proposed to account for the deviation from the Gordon–Taylor in the polymer mixtures^54^
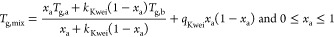
3

Kwei attributed the quadratic term  to the effect of hydrogen bonding forming cross linkages in the polymer mixtures. Studies have used the Kwei equation to fit and explain the *T*_g_-composition relation of hydrogen bonding polymer mixtures.^55−57^ In addition, the Kwei equation can also provide a good fit to the measured *T*_g_ of binary mixtures of molecular liquids.^57−59^ For example, the quadratic term of the Kwei equation used to fit the *T*_g_ of binary blends of trehalose and choline dihydrogen phosphate was attributed to hydrogen bonding.^58^ The Kwei equation has the potential to predict the *T*_g_ of SOA mixtures when the Gordon–Taylor equation fails, particularly in the cases of substantial intermolecular interactions. To our knowledge, the Kwei equation has not been used to predict *T*_g_ of SOA.

**Figure 1:**
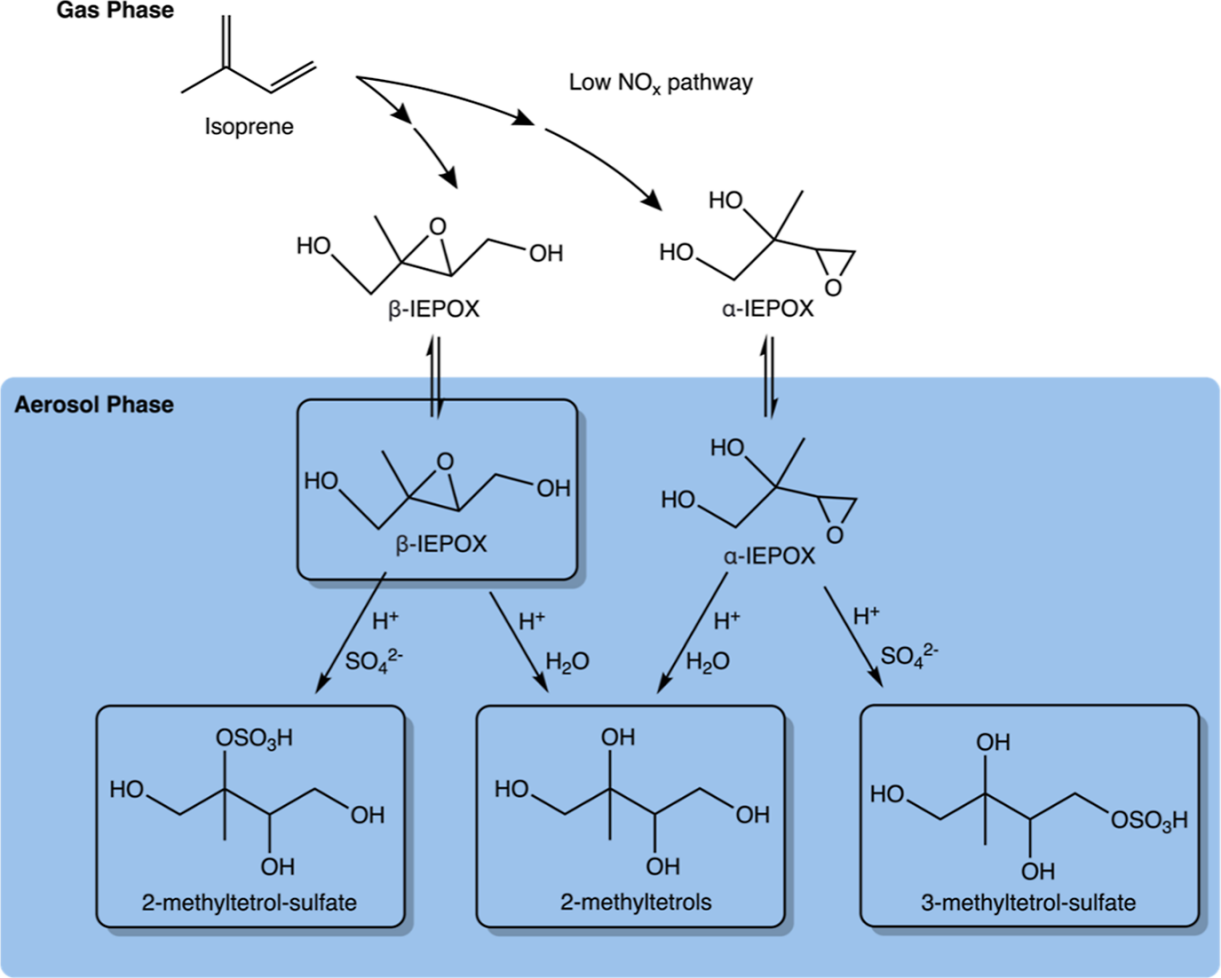
Hydroxyl radical-initiated oxidation of isoprene under low-NO_*x*_ conditions leads to the generation of aerosol-phase β-IEPOX, α-IEPOX, 2-MT, 2-MTS, and 3-MTS.^62,76^ The boxed species are samples used in this study.

Although the *T*_g_ of some SOA mixtures has been studied, the glass transition temperatures of isoprene-derived SOA mixtures have yet to be investigated.^14,15^ Isoprene (2-methyl-1,3-butadiene, C_5_H_8_) is the largest non-methane VOC in the atmosphere and has a short atmospheric lifetime against reactions with hydroxyl radical (OH).^60^ As shown in Figure 1, photooxidation (or OH-initiated oxidation) of isoprene under low-NO_*x*_ conditions forms isoprene epoxydiols (IEPOX, including the predominant β-IEPOX isomers as well as the minor α-IEPOX and δ-IEPOX isomers).^61−63^ Gas-phase IEPOX isomers undergo multiphase reactions once they partition into the aqueous phase of aerosol particles through acid-driven reactive uptake processes.^62^ Reactive uptake of IEPOX yields predominantly 2-methyltetrols (2-MT), 3-methyltetrols (3-MT), and organosulfates, including 2-methyltetrol-sulfate (2-MTS) and 3-methyltetrol-sulfate (3-MTS).^62^ Consequently, isoprene-derived SOA often contains multiple IEPOX-SOA components. Note that 2-MTS and 3-MTS are primarily deprotonated at atmospherically relevant SOA pH.^64^ Though single-component glass transition temperatures of these isoprene-derived SOA constituents have been measured, the mixture *T*_g_ and the interactions between different species are unknown.^20^ Here, we present the first *T*_g_ measurements of *trans*-β-IEPOX-derived SOA mixtures including IEPOX/2-MTS, IEPOX/3-MTS, 2-MT/2-MTS, and 2-MT/3-MTS using BDS under dry conditions. To further investigate and explain the *T*_g_ measurement results, Raman spectroscopy and density functional theory (DFT) simulation of two-molecule systems were used to characterize the level of hydrogen bonding in the studied mixtures.

## Methods

### Sample Preparation

IEPOX, 2-MT, 2-MTS, and 3-MTS were synthesized using published procedures.^65^ The IEPOX isomer used in this study is the atmospherically predominant *trans*-β-IEPOX. In this paper, the *trans*-β-IEPOX sample will be referred to as IEPOX. The concentration of synthesized materials and impurities within the samples was confirmed by nuclear magnetic resonance spectroscopy.^20,65,66^ The purities of IEPOX and 2-MT are 99.9 wt %, the purity of 2-MTS is 60.2 wt %, and the purity of 3-MTS is 57.4 wt %. The bulk of impurities in the 2-MTS and 3-MTS samples is ammonium bisulfate as previously reported.^20^ The mixture samples, 2-MT/2-MTS, 2-MT/3-MTS, IEPOX/2-MTS, and IEPOX/3-MTS, were mixed by volume, assuming equal densities.

### Broadband Dielectric Spectroscopy

A NETZSCH dielectric analysis system, including a dielectric analyzer (DEA 288), a furnace, and IDEs, was used to measure *T*_g_ in this study. The method has been used in previous studies and is described only briefly here.^19,67^

**Figure 2:**
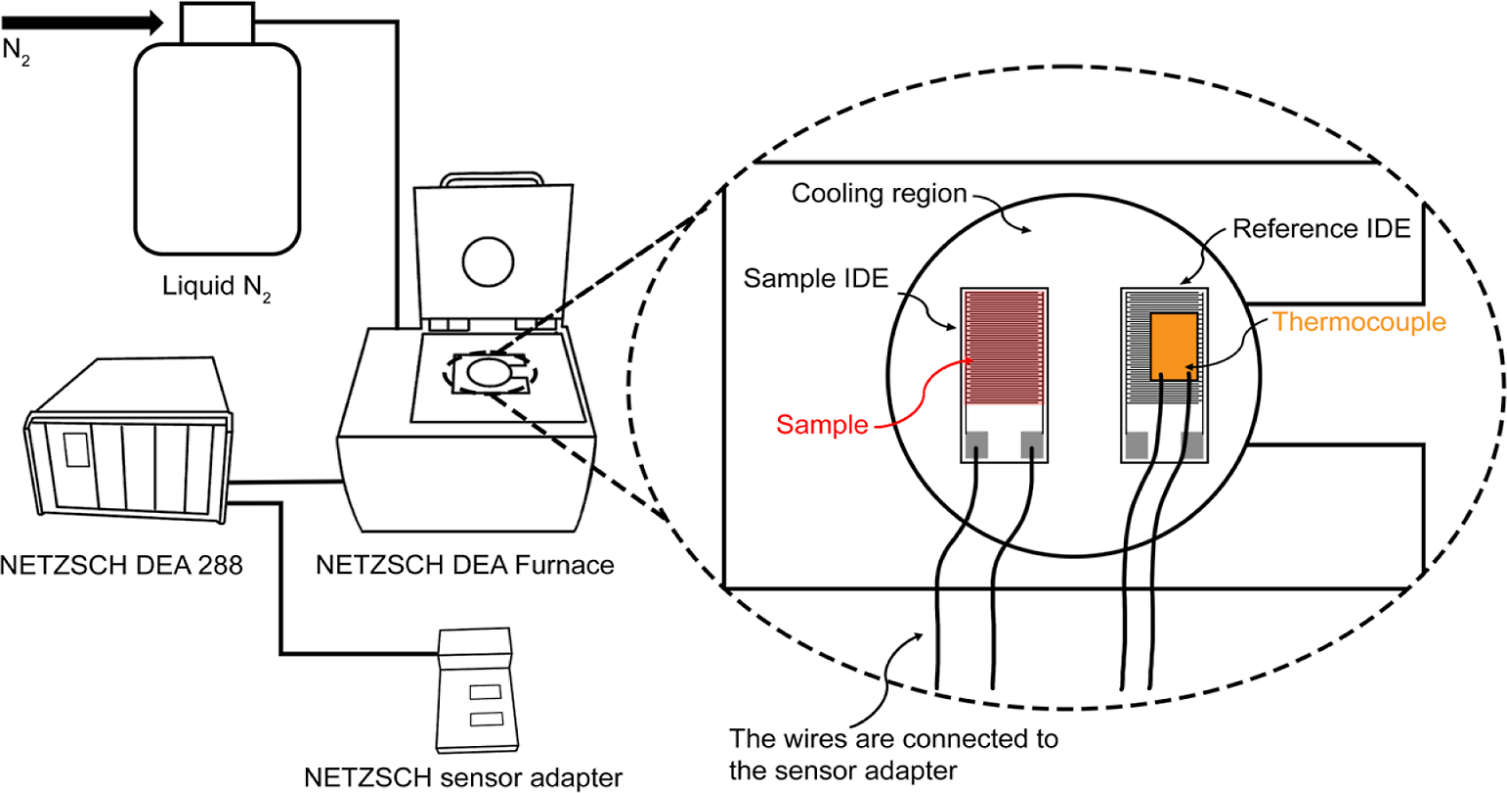
NETZSCH dielectric analyzer DEA 288, sensor adapter box, and the DEA furnace are shown as pictures. The interdigitated electrodes (IDEs) and thermocouple setup inside the heating/cooling chamber of the DEA furnace is shown in line art.

**Figure 3:**
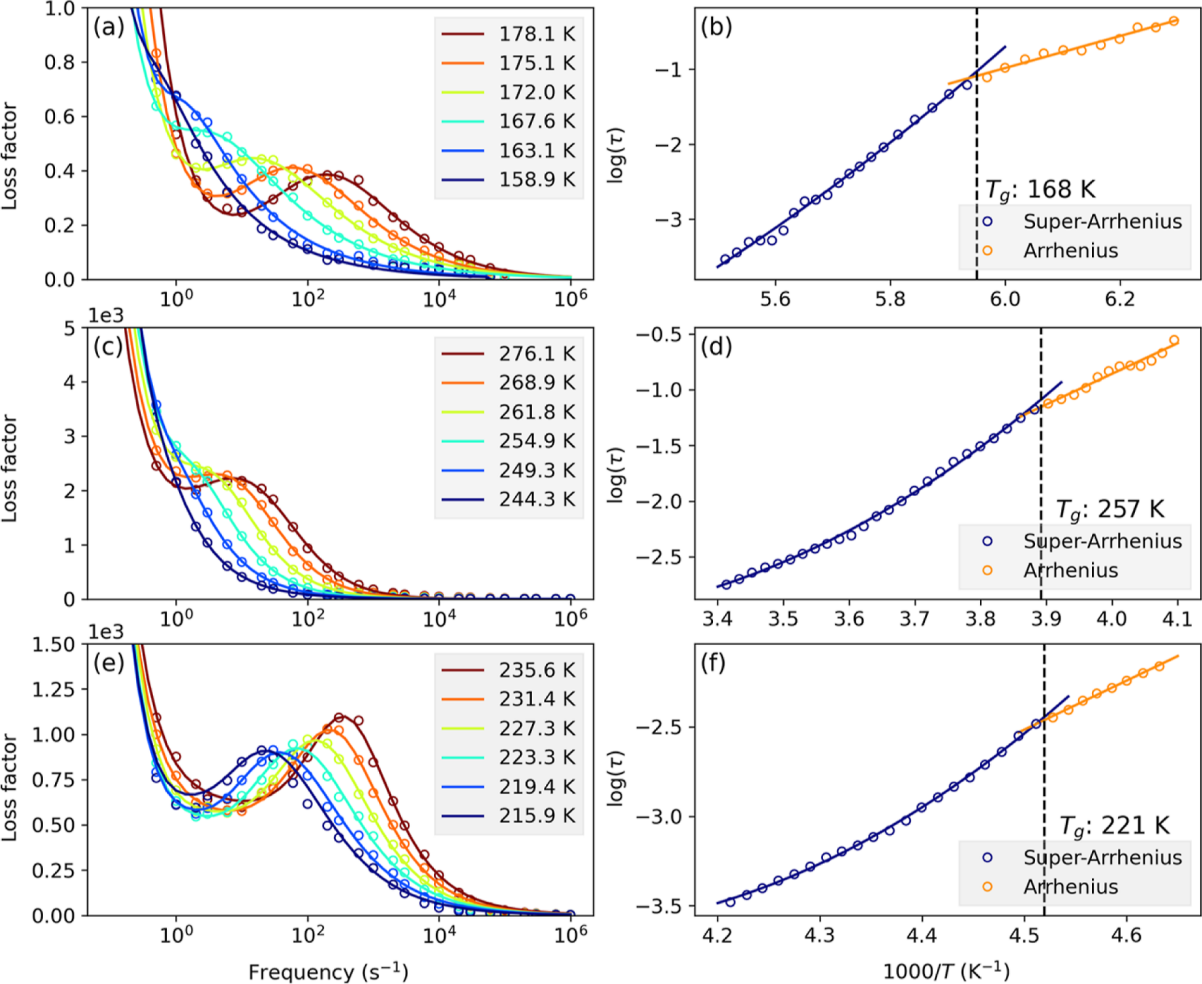
Measured and curve-fitted dielectric spectra of IEPOX are shown in (a). Measured and curve-fitted dielectric spectra of 3-MTS and an IEPOX/3-MTS mixture with 61.2 wt % IEPOX are shown in (c,e), respectively. The open circles are the measured experimental data, and the solid lines are the fitted curves. The log_10_ of relaxation time as a function of the inverse of the temperature of IEPOX, 3-MTS, and the IEPOX/3-MTS mixture with 61.2 wt % IEPOX are shown in (b,d,f). The open circles are the results of the curve fitting a model function to the dielectric loss spectra. The solid lines are fitted to the super-Arrhenius and the Arrhenius regions of the data. The glass transition temperature (*T*_g_) is marked by a vertical dashed line.

During measurement, a thin film of the sample is applied to cover the sample IDE substrate, which is placed into the furnace temperature conditioning chamber, as shown in Figure 2. To measure the temperature, a thermocouple is secured to the reference IDE that is identical to the sample IDE. The reference IDE and thermocouple are also placed in the chamber. The cooling chamber is flushed with nitrogen gas before each measurement to reduce humidity and keep the sample dry during the measurement. The sample IDE and the thermocouple are connected to a dielectric analyzer, which applies an oscillating electric field, scanning through 26 frequencies from 0.5 to 1 × 10^6^ Hz in 30 s. Liquid nitrogen is used to cool the sample at a rate of 5 K min^–1^, from 293 to 123 K. The temperature and frequency-dependent permittivity are recorded by the instrument. Here, the measured permittivity as a function of frequency is referred to as the dielectric function, and the imaginary part of the dielectric function is referred to as the dielectric loss function. For example, Figure 3a shows the measured dielectric loss spectra of IEPOX. The measured dielectric loss spectra of other samples are shown in the Supporting Information.

Next, the dielectric loss function is curve-fitted to the Havriliak–Negami equation to derive dielectric relaxation time, τ.^68−70^ The imaginary part of the Havriliak–Negami equation that is used to fit the measured dielectric loss function is
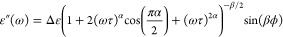
4where
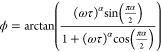
5

ε″ is the imaginary part of the Havriliak–Negami equation. ω is the frequency; τ is the relaxation time of the sample; α and β are the symmetrical broadening and asymmetrical broadening factors of the corresponding peak.^70^ For cases in which a secondary relaxation process exists, the sum of two imaginary parts of Havriliak–Negami equations is used to fit the dielectric function. When impurities (mobile charge carriers) are present, there is a conductivity contribution, which is characterized by an increase in the imaginary part of the complex dielectric function with the decrease in frequency, as shown in Figure 3a.^70^ The equation for the conductivity contribution is
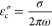
6and σ is the conductivity contribution factor. With a conductivity contribution, the dielectric loss spectra would be the sum of the right-hand side of eqs 4 and 6.

The *T*_g_ is determined by analyzing the change in τ with the change in the inverse of the temperature of a sample. For example, in Figure 3b, the log_10_ of τ is plotted as a function of the inverse of the temperature for IEPOX. As Figure 3b illustrates, as the temperature decreases, there is a transition in the log_10_ τ versus the inverse of the temperature relationship, which reflects the transition from the super-Arrhenius behavior of the supercooled liquids to the Arrhenius behavior of the glass upon cooling below *T*_g_.^19,20,71−73^ To identify *T*_g_, every two adjacent data points of the temperature-dependent τ are selected as the intersections to divide the data into two regions, the super-Arrhenius region and the Arrhenius region. The super-Arrhenius region of the data is fitted with
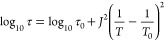
7

While the Arrhenius region is fitted with
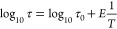
8

The *R*^2^ of the fit of the two regions was summed for each intersection, and the intersection with the highest summed *R*^2^ was identified as the *T*_g_, as shown in Figure 3b. For comparison, measured dielectric spectra and the log_10_ τ as a function of inverse temperature are shown in Figure 3c,d for 3-MTS, and in Figure 3e,f for the IEPOX/3-MTS mixture with 61.2 wt % IEPOX, respectively.

Given that 2-MTS, 3-MTS, and mixtures contain ammonium bisulfate as an impurity of the synthesis procedure, the Gordon–Taylor equation was applied to these samples to determine the true *T*_g_ of the organic components.^20,65,66^ The calculation is based on the measured amount of ammonium bisulfate, a *T*_g_ of 220 ± 3 K for pure ammonium bisulfate, and a Gordon Taylor constant *k*_GT_ of 0.75 ± 0.25.^20^ While this approximation introduces an added uncertainty to our determined *T*_g_ values, to the best of our knowledge, a more rigorous method for accounting for the additional component is unavailable at this time. Corrected *T*_g_ is calculated for pure 2-MTS, pure 3-MTS, and the organic mixtures after accounting for the absence of ammonium bisulfate.

The uncertainty of measured *T*_g_ was estimated while determining the *T*_g_ from temperature-dependent dielectric relaxation time τ. A sensitivity analysis of choosing different temperature ranges for finding *T*_g_ shows that the uncertainty of the measured *T*_g_ is 1 to 3 K, which is a relatively small uncertainty range compared with previous studies. The more detailed process of determining *T*_g_ uncertainty is shown in the Supporting Information. Additionally, the difference between the temperature of the sample and the temperature measured by the thermocouple could be up to 2 K, as previously reported, adding an additional 2 K as uncertainty.^19^ The uncertainty of corrected *T*_g_ was calculated through error propagation while correcting the *T*_g_ of samples with impurities.

### Raman Spectroscopy

The Raman spectra of 2-MT/3-MTS mixtures were recorded with a Horiba XploRA One Raman microscope combined with an LTS120 Linkam cooling stage. All spectra were recorded using a 532 nm excitation laser, a 10× microscope objective, and a spectral range of 400–4000 cm^–1^. For each measurement, a small drop of the sample is transferred to a hydrophobic microscope slide (coated with Rain-X Original Water Glass Repellant) using a micropipette. This slide is placed in the Linkam cooling stage, which was then sealed. A constant flow of dry nitrogen gas at 0.5 standard liters per minute is passed through the stage to prevent water condensation onto the sample during Raman measurement. Consequently, the samples do not contain water. The cooling stage temperature is maintained at −40 °C, while the Raman spectra are recorded to acquire spectra of samples in the glassy phase. The collected spectra are baseline corrected using the ModPoly algorithm, which is based on a modification to polynomial fitting.^74^ To obtain the wavenumber of the O–H stretching bands, the Raman spectra between 2600 and 3700 cm^–1^ are curve-fitted using a Gaussian–Lorentzian peak profile. The C–H stretching bands, the N–H stretching band, and the O–H stretching bands are fitted.^75,76^

### DFT Simulations of Hydrogen Bonds within Binary Complexes

The Gordon–Taylor approximation does not account for interactions between components.^58^ Consequently, when significant intermolecular interactions occur, observed glass transition temperatures in mixed systems deviate from Gordon–Taylor predictions. In this study, DFT was used to model the hydrogen bonding in two homogeneous binary complexes, 2-MT/2-MT and 3-MTS/3-MTS, as well as in a heterogeneous binary complex, 2-MT/3-MTS. In the simulation, the geometry of the three two-molecule systems is first optimized using ABCluster software with the xTB method.^77,78^ Next, geometry optimization and energy calculations are performed using Gaussian 16 with B3LYP-D3 dispersion corrected functional and 6-31++g(2d,2p) basis set.^79^ Counterpoise correction was applied in the interaction energy calculation. The functional and the basis set are chosen for their efficiency and reasonable accuracy. The outputs are visualized in VMD software to determine the number of hydrogen bonds with a distance and angle cutoff value.^80^ The distance cutoff (maximum distance between the proton donor and the acceptor) is set to 3.2 Å, and the angle cutoff (minimum angle made by the proton donor, the hydrogen atom, and the proton acceptor) is set to 130°, based on the definition of strong hydrogen bonds in Desiraju & Steiner.^81^ The interaction energy is calculated with the supermolecular approach. The interaction energy *E* between two molecules, A and B, is calculated as
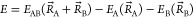
9where  and  are coordinates of the atoms in molecules A and B, respectively.^82,83^*E*_A_ and *E*_B_ are the unrelaxed energy of molecules A and B, respectively. *E*_AB_ is the relaxed energy of the optimized hydrogen-bonded binary complex. *E* includes not only hydrogen bonding energy but also van der Waals interactions energy. However, since hydrogen bonding energy is much stronger than van der Waals interactions, the interaction energy *E* calculated here can be used as a slightly overestimated approximation of the hydrogen bond energy.

## Results and Discussion

### Glass Transition Temperature Measurements

The measured glass transition temperatures of individual IEPOX-derived SOA constituents are listed in Table 1. Glycerol is included as a standard. Within the uncertainty in the measurements, the *T*_g_ value of pure glycerol, 2-MT, and IEPOX agree with the literature.^20^ Following the correction for ammonium bisulfate as discussed above, the *T*_g_ value of 2-MTS also agrees with the literature.^20^ This study is the first report of *T*_g_ of 3-MTS. The difference between the corrected *T*_g_ of 2-MTS and 3-MTS is within the measurement uncertainty. The 2-MTS and 3-MTS are isomers with a difference in the methyl group position. Thus, it was expected that the *T*_g_ of 2-MTS and 3-MTS would be similar.

**Table 1:** *T*_g_ of Single Organic Samples

sample	organic (wt %)^a^	measured *T*_g_ (K)	corrected *T*_g_ (K)^b^	literature (K)^20^
glycerol	100.0	193 ± 2	N/A	192 ± 2
IEPOX	100.0	168 ± 3	N/A	166 ± 2
2-MT	100.0	231 ± 3	N/A	230 ± 2
2-MTS	60.2	256 ± 3	274 ± 7	276 ± 15
3-MTS	57.4	257 ± 4	278 ± 9	N/A

a Remaining wt % is ammonium bisulfate.

b Corrected for ammonium bisulfate impurities.

**Figure 4:**
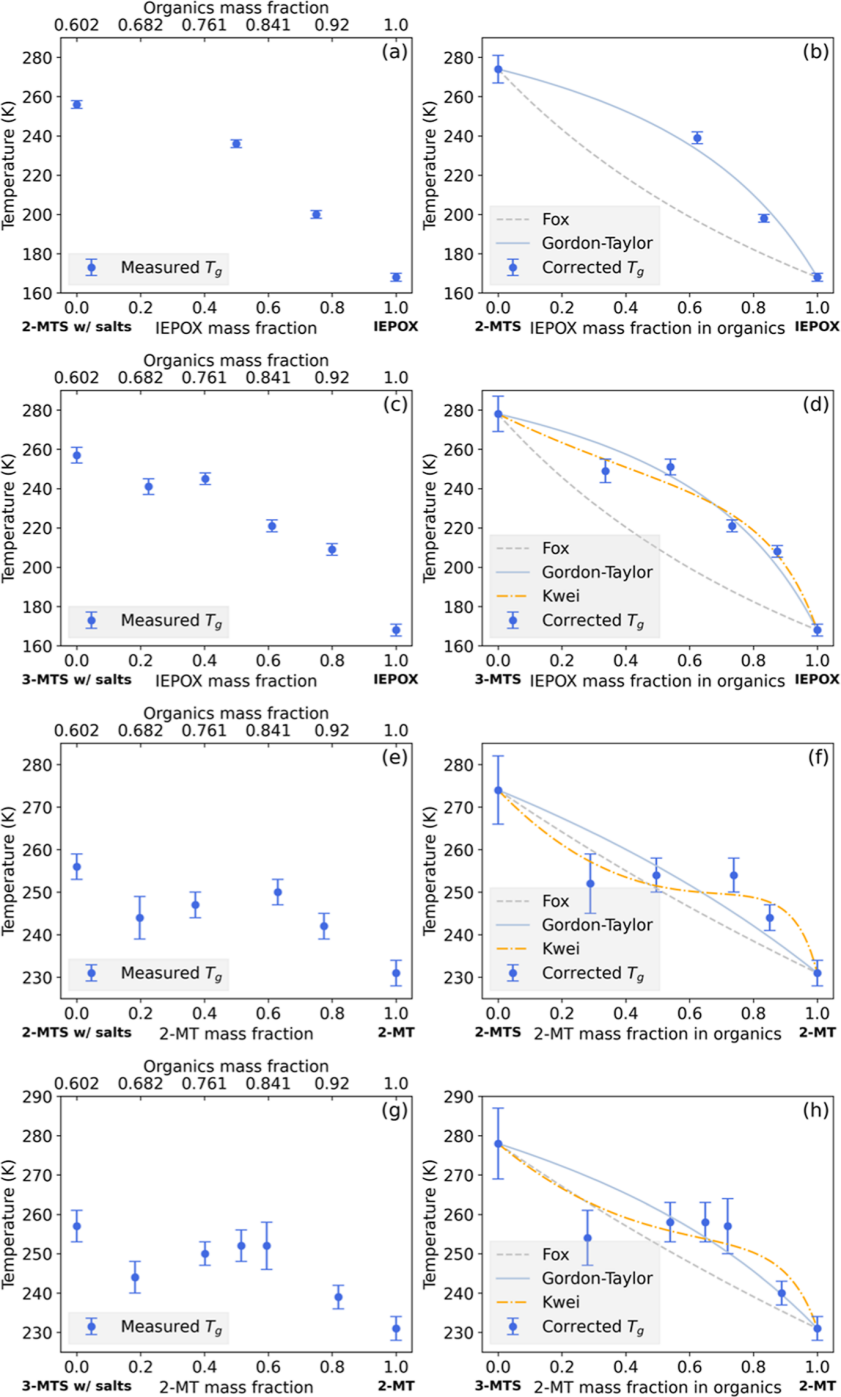
Measured *T*_g_ at different IEPOX mass fractions in IEPOX/2-MTS and IEPOX/3-MTS mixtures are shown in (a,c), respectively. Corrected *T*_g_ at different IEPOX mass fractions in IEPOX/2-MTS and IEPOX/3-MTS mixtures after removing ammonium bisulfate effects are shown in (b,d), respectively. Measured *T*_g_ at different 2-MT mass fractions in 2-MT/2-MTS and 2-MT/3-MTS mixtures are shown in (e,g), respectively. *T*_g_ at different 2-MT mass fractions in 2-MT/2-MTS and 2-MT/3-MTS mixtures after correction are shown in (f,h), respectively.

The measured and corrected glass transition temperatures for mixtures are listed in Table 2. Figure 4a shows the measured glass transition temperatures of IEPOX/2-MTS mixtures. The measured glass transition temperatures of the IEPOX/2-MTS mixtures are between the *T*_g_ of IEPOX and 2-MTS, as shown in Figure 4b. Theoretical *T*_g_ predicted by Gordon–Taylor and Fox equations are also shown in Figure 4b. Given the limited number of data points, it was not possible to predict *T*_g_ in Figure 4a using the Kwei equation. Figure 4b demonstrates that the Fox equation underestimates and fails to predict the *T*_g_ for the IEPOX/2-MTS mixtures, while the Gordon–Taylor equation captures the shape of the *T*_g_-composition relationship for this system with an *R*^2^ of 0.99.

**Table 2:** *T*_g_ of Organic Mixture Samples

sample	organics (wt %)^a^	IEPOX (wt %)	IEPOX (wt % in organics)	measured *T*_g_ (K)	corrected *T*_g_ (K)^b^
IEPOX/2-MTS Mixtures					
mix 1	80.1	50.0	62.4	236 ± 3	239 ± 3
mix 2	90.1	75.0	83.3	200 ± 3	198 ± 2
IEPOX/2-MTS Mixtures					
mix 1	67.0	22.5	33.6	241 ± 4	249 ± 6
mix 2	74.5	40.2	54.0	245 ± 3	251 ± 4
mix 3	83.5	61.2	73.3	221 ± 3	221 ± 3
mix 4	91.0	80.0	87.5	209 ± 3	208 ± 3
2-MT/2-MTS Mixtures					
mix 1	68.0	19.7	28.9	244 ± 5	252 ± 7
mix 2	75.0	37.1	49.5	247 ± 3	254 ± 4
mix 3	85.3	63.0	73.9	250 ± 3	254 ± 4
mix 4	91.0	77.5	85.1	242 ± 3	244 ± 3
2-MT/2-MTS Mixtures					
mix 1	65.2	18.3	28.0	244 ± 4	254 ± 7
mix 2	74.5	40.2	53.9	250 ± 3	258 ± 5
mix 3	79.3	51.5	64.9	252 ± 4	258 ± 5
mix 4	82.8	59.6	71.9	252 ± 6	257 ± 7
mix 5	92.3	82.0	88.8	239 ± 3	240 ± 3

a Remaining wt % is ammonium bisulfate.

b Corrected for ammonium bisulfate impurities.

In addition, the measured *T*_g_ of IEPOX/3-MTS, 2-MT/2-MTS, and 2-MT/3-MTS mixtures are shown in Figure 4c,e,g, respectively. The Fox equation, curve-fitted Gordon–Taylor equation, and curve-fitted Kwei equation for IEPOX/3-MTS, 2-MT/2-MTS, and 2-MT/3-MTS mixtures are shown in Figure 4d,f,h, respectively. The curve-fit results are shown in Table 3. Similar to the IEPOX/2-MTS mixture, the IEPOX/3-MTS *T*_g_ value follows the Gordon–Taylor relation with an *R*^2^ value of 0.97. In contrast, the Gordon–Taylor equation does not provide good fits to the 2-MT/2-MTS and 2-MT/3-MTS mixtures, with an *R*^2^ value of 0.74 for the 2-MT/2-MTS mixture and 0.77 for the 2-MT/3-MTS mixture. Figure 4f,h shows that the Gordon–Taylor equation cannot capture the S-shaped *T*_g_-composition relation for 2-MT/2-MTS and 2-MT/3-MTS mixtures. The Kwei equation provides good fits to the *T*_g_ of IEPOX/3-MTS, 2-MT/2-MTS, and 2-MT/3-MTS, with an *R*^2^ value of 0.98, 0.93, and 0.88, respectively. It can be inferred that since the *T*_g_ difference between pure components of the binary mixture is larger in the IEPOX/2-MTS and IEPOX/3-MTS, the Gordon–Taylor term (the first term of the Kwei equation) dominates in the *T*_g_-composition relation of these two mixtures, and thus, the Gordon–Taylor equation provides an adequate fit. When the *T*_g_ difference between the pure components of binary mixtures is smaller, as is the case in the 2-MT/2-MTS and 2-MT/3-MTS mixtures, the quadratic term in the Kwei equation becomes important, and the Kwei equation is needed to provide a better *T*_g_ prediction for these mixtures. Our findings agree with our previous observation that the *T*_g_ of binary mixtures of two components with similar *T*_g_ can be non-linear, while the *T*_g_ of SOA is affected predominantly by components that have large differences in *T*_g_.^20^

**Table 3:** Curve Fitting Results

		Gordon–Taylor equation		Kwei equation		
mixture	*T*_g_ difference (K)	*k*_GT_	*R*^2^	*k*_Kwei_	*q*_kwei_	*R*^2^
IEPOX/2-MTS	106 ± 8	2.62	0.99	N/A	N/A	N/A
IEPOX/3-MTS	110 ± 9	2.91	0.97	4.75	–56.77	0.98
2-MT/2-MTS	43 ± 8	1.39	0.74	8.58	–72.42	0.93
2-MT/3-MTS	47 ± 9	1.79	0.77	6.38	–60.5	0.88

Since IEPOX, 2-MT, 2-MTS, and 3-MTS molecules all have multiple hydroxy groups which can form hydrogen bonding, we attribute the quadratic term in the Kwei equation to changes in the level of hydrogen bonding in the mixed systems as compositions change, as originally proposed by Kwei.^54^ For example, in the 2-MT/3-MTS system, since mixing results in hydrogen bonding between the 2-MT and 3-MTS molecules in the system (represented by the *q* term in the Kwei equation), the S-shaped trend in *T*_g_ deviates negatively from the Gordon–Taylor equation (as seen in Figure 4h). We used Raman spectroscopy and DFT structure optimization to further assess the extent of hydrogen bonding in the 2-MT/3-MTS mixture systems. For clarity, we note a recent study provides evidence that under higher humidity conditions, 2-MTS and 3-MTS in aqueous solution become deprotonated.^64^ However, since no water has been added to the liquid mixtures here, deprotonation is unlikely to factor into the hydrogen bonding in these samples.

### Raman Characterization of Hydrogen Bonding

Previous studies have linked hydrogen bonding and hydrogen bonding networks to the *T*_g_ for molecular hydrocarbons.^84−89^ A study using Raman spectroscopy has shown that *T*_g_ is strongly associated with the strength and density of hydrogen bonding for various saccharides.^85^ It was also shown that for polyhydric alcohol, *T*_g_ is more dependent on the number of hydroxy groups rather than the number of carbon atoms.^87^ Later studies investigated various mixtures and determined that *T*_g_ is linearly dependent on the number of hydroxy groups for polyol solutions and many dry polyol mixtures.^88^ Given that 2-MT and 3-MTS all have multiple hydroxy groups, the *T*_g_ of their mixtures could be strongly dependent on hydrogen bonding.

**Figure 5:**
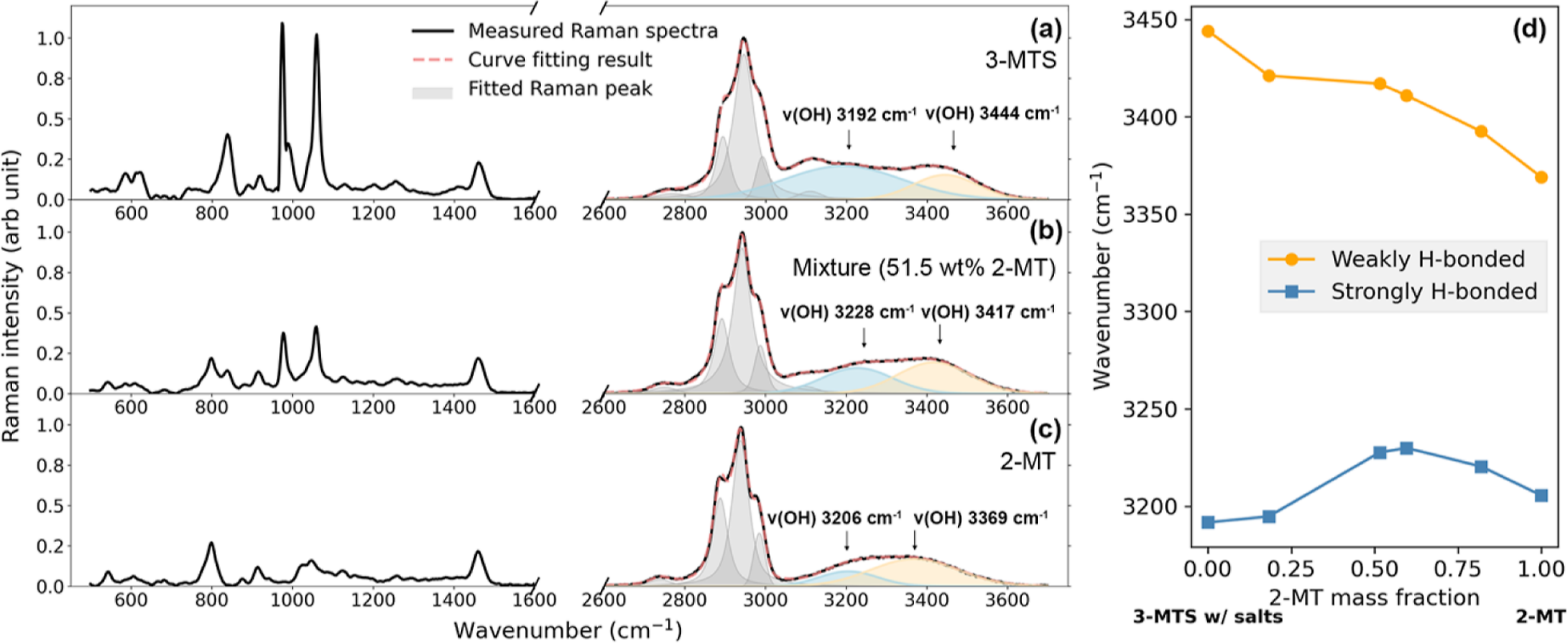
Recorded and curve-fitted Raman spectra of 3-MTS, a 2-MT/3-MTS mixture, and 2-MT are shown in (a–c), respectively. (d) shows the wavenumber of the OH stretching peaks of different 2-MT/3-MTS mixtures. The orange line corresponds to the orange peak in the Raman spectra representing weakly hydrogen-bonded peaks, while the blue line corresponds to the blue peak in the Raman spectra representing strongly hydrogen-bonded peaks.

Raman spectra of the 2-MT sample, 3-MTS sample, and 2-MT/3-MTS mixtures were recorded to analyze the extent of hydrogen bonding in these samples. The Raman spectrum of 3-MTS is shown in Figure 5a. The Raman spectra of a 2-MT/3-MTS mixture (with 51.5 wt % 2-MT) and pure 2-MT are shown in Figure 5b,c, respectively. The measured Raman spectra are shown as black solid lines, while the curve-fitted Raman spectra from 2600 to 3700 cm^–1^ are shown as red dashed lines. The individual curve-fitted peaks are shown as thin lines with shaded peak areas. The Raman spectra of 3-MTS and 2-MT match well with previously published spectra, confirming the identities of the samples.^76^ The detailed figures of the decomposition of Raman spectra from 2600 to 3700 cm^–1^ are shown in the Supporting Information.

The O–H stretching bands were observed and analyzed in all measured Raman spectra. Hydrogen bonding decreases the vibrational frequency of O–H stretching, and the O–H Raman peak with a lower wavenumber is associated with stronger hydrogen bonding.^90^ Therefore, the extent of hydrogen bonding can be interpreted from the decomposition of the Raman O–H stretching bands. In Figure 5a, characteristic peaks include two O–H stretching bands, one at ∼3200 cm^–1^ associated with the strongly hydrogen-bonded hydroxy group in the molecule and a second at ∼3400 cm^–1^ associated with the weakly hydrogen-bonded hydroxy group.^91,92^ The curve-fitted peak position of both the weaker and stronger O–H stretching peaks for mixtures at different 2-MT mass fractions is shown in Figure 5d. The wavenumber of weakly hydrogen-bonded O–H peak decreases from 3444 to 3369 cm^–1^ over the range of 0 to 1 2-MT mass fraction, showing that the weak hydrogen bonds strengthen as the 2-MT weight fraction increases. However, for the strongly hydrogen-bonded O–H peak, a similar trend with the mass fraction is not observed. In fact, moving from 0 to 1 2-MT mass fraction, the wavenumber of strongly hydrogen-bonded O–H peak increased from 3192 to 3130 cm^–1^ and then decreased to 3206 cm^–1^. The strong hydrogen bonds in the 3-MTS sample are the strongest, and the evenly mixed 2-MT/3-MTS mixtures have the weakest strong hydrogen bonds. The trend of the wavenumber of strongly hydrogen-bonded peaks is evidence that hydrogen bonding is related to *T*_g_ in our samples. The weakening of the strong hydrogen bonding in 2-MT/3-MTS mixtures could potentially explain the negative deviation of the mixtures’ *T*_g_-composition relation as represented by the quadratic term in the Kwei equation. However, a complication is that hydrogen bonding between like molecules also occur. Further investigation is needed to confirm the trend between hydrogen bonding and *T*_g_.

### Simulation of Hydrogen Bonding in Selected Binary Complexes

**Figure 6:**
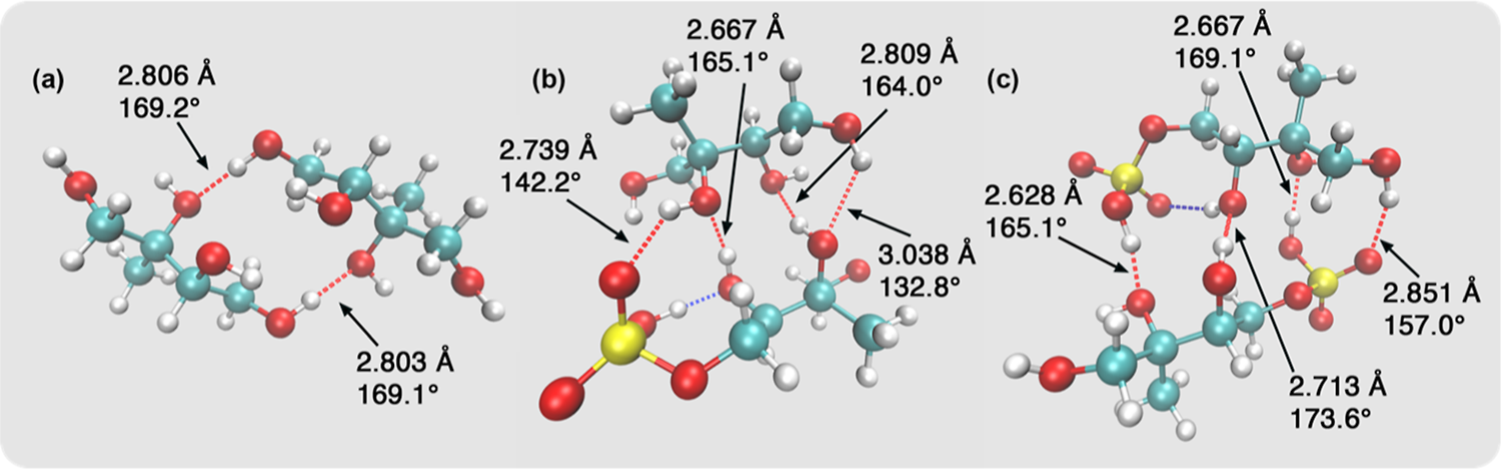
Optimized geometry of (a) 2-MT/2-MT binary complex, (b) 2-MT/3-MTS binary complex, and (c) 3-MTS/3-MTS complex. Red dashed lines represent intermolecular hydrogen bonds, and blue dashed lines represent intramolecular hydrogen bonds. The donor-to-acceptor distance and the angle of the intermolecular hydrogen bonds are marked in the figure. The hydrogen bonds are identified based on a distance cutoff of 3.2 Å and an angle cutoff of 130°.

To further demonstrate the extent of the hydrogen bonding in the sample, geometry optimizations of binary complexes using DFT were produced and inspected. While the binary complexes are simple systems, they could illustrate the high extent of intermolecular interactions between the molecules. Figure 6a shows the optimized structure and hydrogen bonding in a 2-MT/2-MT complex. Figure 6b,c shows the optimized structure and hydrogen bonding in a 2-MT/3-MTS complex and a 3-MTS/3-MTS complex, respectively. Although organosulfates are deprotonated in atmospheric conditions, here we used the protonated structure of 3-MTS in our idealized simulations of the binary complex without temperature and solvation effects.^64^Table 4 shows the result of the simulation of hydrogen bonding in a 2-MT/2-MT complex, a 3-MTS/3-MTS complex, and a 2-MT/3-MTS complex. As shown in Table 4, the 2-MT/2-MT complex has two hydrogen bonds with the lowest interaction energy of −88.03 kJ/mol; the 2-MT/3-MTS complex has four hydrogen bonds with an interaction energy of −120.71 kJ/mol; and the 3-MTS/3-MTS complex has four hydrogen bonds with the highest interaction energy at 183.30 kJ/mol. Note that the intramolecular hydrogen bonds are not included in the interaction energy calculations in Table 4. The optimized 3-MTS/3-MTS complex and 2-MT/3-MTS complex have more intermolecular hydrogen bonds than the optimized 2-MT/2-MT complex because the oxygen atoms in the sulfate group of the 3-MTS molecule act as proton acceptors. However, in systems with more than two molecules, other hydroxy groups in the 2-MT/2-MT complex could potentially form more hydrogen bonds. As a result, the average intermolecular hydrogen bonding energy might be a more accurate representation of the strength of hydrogen bonding in the bulk system. The average intermolecular hydrogen bond energy of the 2-MT/2-MT complex is about −44.02 kJ/mol, the average intermolecular hydrogen bond energy of the 2-MT/3-MTS complex is the weakest at −30.18 kJ/mol, and the average intermolecular hydrogen bond energy of the 3-MTS/3-MTS complex is the strongest at −45.83 kJ/mol. Despite having an equal number of hydrogen bonds, the 2-MT/3-MTS complex exhibits significantly lower interaction energy than the 3-MTS/3-MTS complex. This is attributed to the smaller angle of the hydrogen bonds formed in the 2-MT/3-MTS complex, as seen in Figure 6. In addition, according to Figure 6b,c, 3-MTS tends to form intramolecular hydrogen bonding due to the added flexibility of the sulfate group. The dynamic balance of intramolecular and intermolecular hydrogen bonds formed by the 3-MTS molecule and the dynamic balance of the stronger and weaker hydrogen bonds between 2-MT and 3-MTS molecules could potentially explain the decrease of hydrogen bonding strength in the 2-MT/3-MTS mixtures as identified by the Raman spectroscopy.

**Table 4:** DFT Simulation Results Using the B3LYP-D3 Functional, 6-31++g(2d,2p) Basis Set

binary complex	number of intermolecular H-bonds^a^	interaction energy (kJ/mol)	average intermolecular H-bond energy (kJ/mol)	number of intramolecular H-bonds^a^	number of total H-bonds
2-MT/2-MT	2	–88.03	–44.02	0	2
2-MT/3-MTS	4	–120.71	–30.18	1	5
3-MTS/3-MTS	4	–183.30	–45.83	1	5

a Hydrogen bonds determined from a distance cutoff of 3.2 Å and an angle cutoff of 130°.

This geometry optimization using DFT is limited to binary complexes, but the results can provide a qualitative comparison of the hydrogen bonding among our studied systems. Further work involving molecular simulations containing many more molecules than the simplified binary complexes may provide further insights into the connection between molecular interactions and glass transition temperatures.

## Conclusions

In this study, we present measurements of the *T*_*g*_ of mixtures of IEPOX-derived SOA compounds measured by BDS. For comparison, calculations of *T*_g_ for the mixtures were performed using Fox, Gordon–Taylor, and Kwei equations in the literature to test the validity of the equations in predicting *T*_g_ values in mixed systems. The agreement between measurements and the Fox equation was generally poor. The observed *T*_g_ values of IEPOX/2-MTS and IEPOX/3-MTS mixtures were in good agreement with the Gordon–Taylor equation. However, 2-MT/2-MTS and 2-MT/3-MTS mixtures exhibit S-shaped behavior in *T*_g_, which was not successfully predicted by the Gordon–Taylor equation. The Kwei equation provides a better fit to the observed *T*_g_ values of IEPOX/3-MTS, 2-MT/2-MTS, and 2-MT/3MTS mixtures by accounting for intermolecular forces, including hydrogen bonding. When the Gordon–Taylor equation does not work, the Kwei equation can be used instead to calculate the *T*_g_ values of mixtures for compounds with strong intermolecular forces.

Furthermore, we speculate that the observed S-curved trend in *T*_g_ with mixture composition is caused by changes in hydrogen bonding level due to changing composition, which we further explored using Raman spectroscopy and DFT. The Raman spectra and the DFT calculations on pairwise molecular groups provide evidence of the high degree of hydrogen bonding present in the mixed systems. These inferred and modeled intermolecular forces appear to give rise to the observed nonlinear changes in *T*_g_ with the composition of IEPOX-derived SOA constituents measured here. Molecular dynamics studies of systems larger than our studied binary complex clusters are needed to quantify the impact of these interactions on *T*_g_ of SOA systems and to assess the overall implications for atmospheric SOA.

## Supporting Information Available

Glass transition temperatures of organic mixtures from IEPOX-derived SOA, experimental details for BDS, and all measured Raman spectra from 2600 to 3700 cm^–1^ (PDF)
