# Supplementary material for: Glass Transition Temperatures of Organic Mixtures from Isoprene Epoxydiol-Derived Secondary Organic Aerosol
Source: J Phys Chem A. 2023 May 2;127(18):4125–36. doi: 10.1021/acs.jpca.2c08936 (PMC10863072; doi:10.1021/acs.jpca.2c08936)
Supplement: Supplementary file 1 — jp2c08936_si_001.pdf [file jp2c08936_si_001.pdf]

Supporting Information: Glass Transition Temperatures of Organic Mixtures from Isoprene Epoxydiol (IEPOX)-Derived Secondary Organic Aerosol

for submission to J. Phys. Chem. A

Bo Chen,[1] Jessica A. Mirrielees,[2] Yuzhi Chen,[3] Timothy B. Onasch,[4] Zhenfa Zhang,[3] Avram Gold,[3] Jason D. Surratt,[3, 5] Yue Zhang,[1] and Sarah D. Brooks[1*]

1. Department of Atmospheric Sciences, Texas A&M University. Eller O&M Building, 1204, 3150 TAMU, 797 Lamar St, College Station, Texas 77843
2. Department of Chemistry, University of Michigan, 930 N University Ave, Ann Arbor, Michigan 48104
3. Gillings School of Global Public Health, Department of Environmental Sciences and Engineering, University of North Carolina at Chapel Hill, 170 Rosenau Hall, Campus Box #7400, 135 Dauer Drive, Chapel Hill, North Carolina 27599
4. Aerodyne Research, Inc. 45 Manning Rd, Billerica, Massachusetts 01821
5. College of Arts and Sciences, Department of Chemistry, University of North Carolina at Chapel Hill, Campus Box #3290, 125 South Rd, Chapel Hill, North Carolina 27599

*To whom correspondence should be addressed: sbrooks@tamu.edu

Process of determining T_g uncertainty in Broadband Dielectric Spectroscopy (BDS) analysis

As stated in the main text, a program is used to find the T_g by finding the transition between the Super Arrhenius region and the Arrhenius region in the $\log_{10}$ of τ versus the inverse of the temperature relationship. Choosing a slightly different temperature range as input for the program may lead to 1-3K of difference in outputted T_g result. For example, for IEPOX, choosing different temperature ranges as input could lead to a difference in resulted in $T_{g,}$ as shown in Figure S1. Figure S1 shows T_g determined from four 1000/T ranges. Figure S1a shows the 1000/T range from 5.4 K^{-1} to 6.3 K^{-1} with a T_g result of 168K; Figure S1b shows the 1000/T range from 5.5 K^{-1} to 6.3 K^{-1} with a T_g result of 168K; Figure S1c shows the 1000/T range from 5.6 K^{-1} to 6.3 K^{-1} with a T_g result of 169K; S1d shows the 1000/T range from 5.7 K^{-1} to 6.1 K^{-1} with a T_g result of 171K. Since more temperature ranges lead to a result of 168 K, 168 K is chosen to be the measured T_g of IEPOX, with ± 3K as uncertainty.

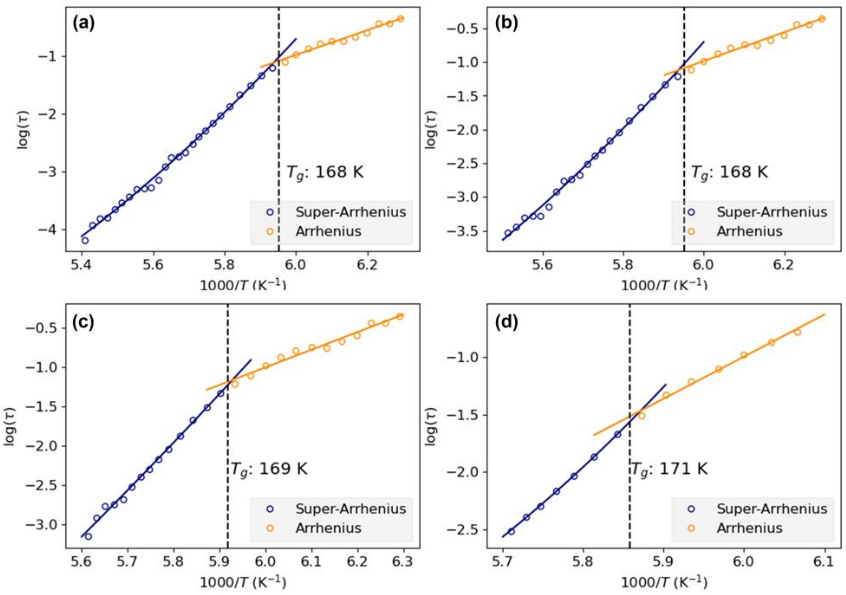

Figure S1 Choosing different temperature range for analysis result in slightly different T_g.

Supporting Figures for BDS Analysis

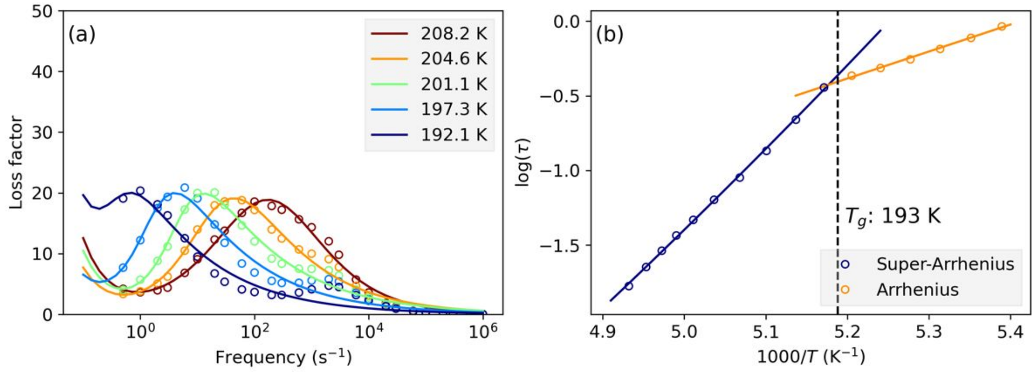

Figure S2 (a) Glycerol Dielectric Spectra and (b) relaxation time-temperature relation

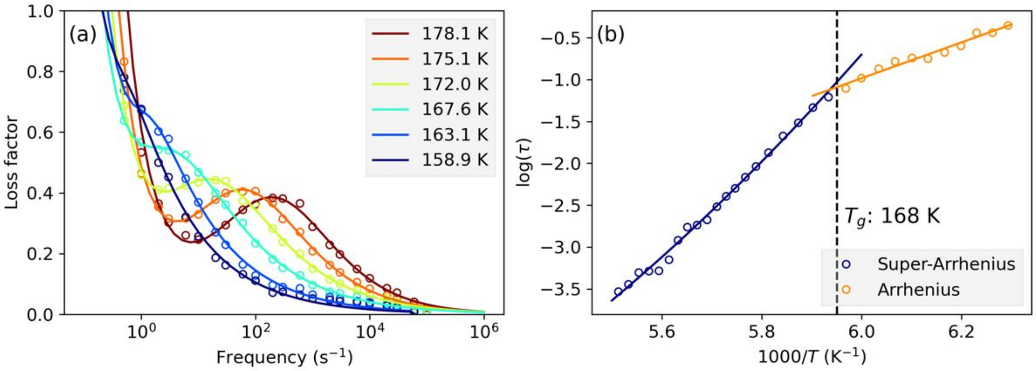

Figure S3 (a) IEPOX Dielectric Spectra and (b) relaxation time-temperature relation

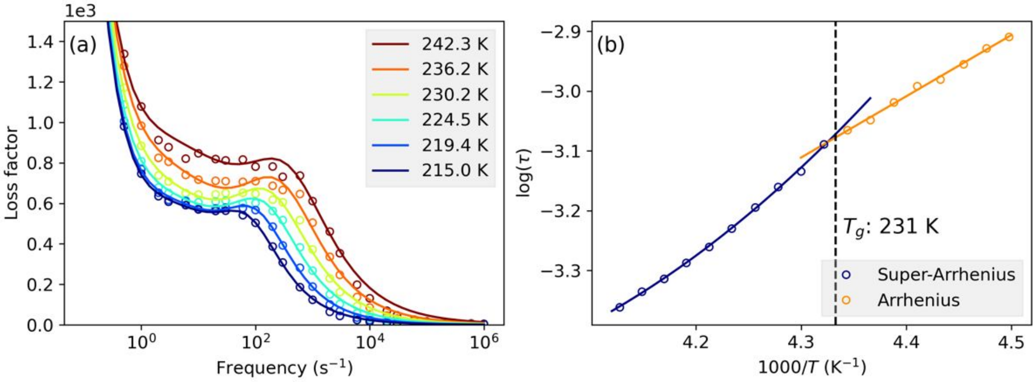

Figure S4 (a) 2-MT Dielectric Spectra and (b) relaxation time-temperature relation

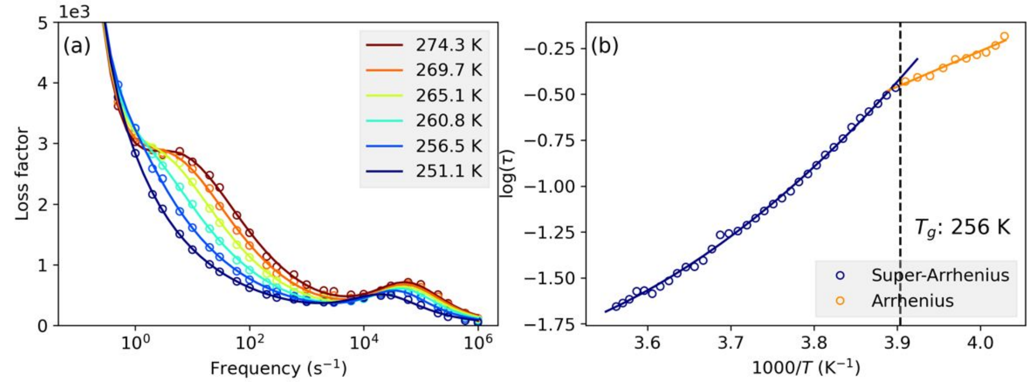

Figure S5 (a) 2-MTS Dielectric Spectra and (b) relaxation time-temperature relation

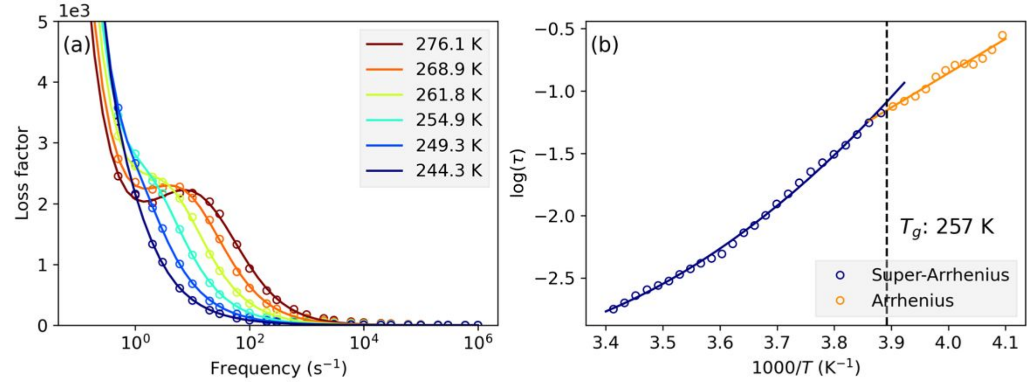

Figure S6 (a) 3-MTS Dielectric Spectra and (b) relaxation time-temperature relation

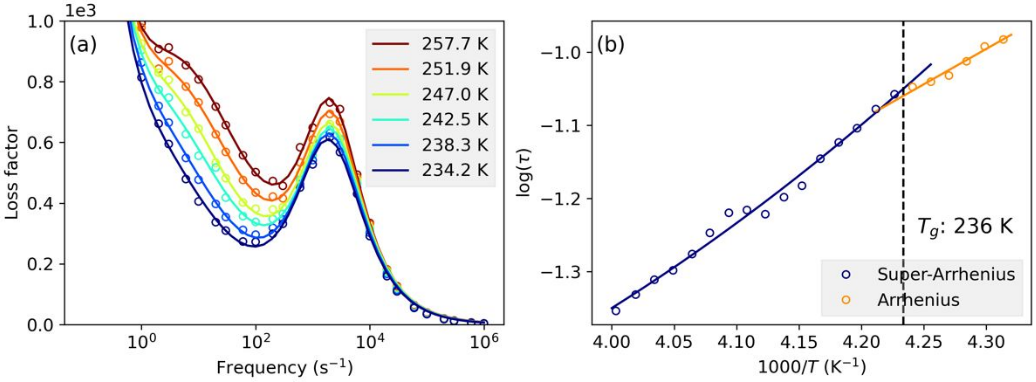

Figure S7 (a) IEPOX/2-MTS mixture (with 50wt% of IEPOX) Dielectric Spectra and (b) relaxation time-temperature relation

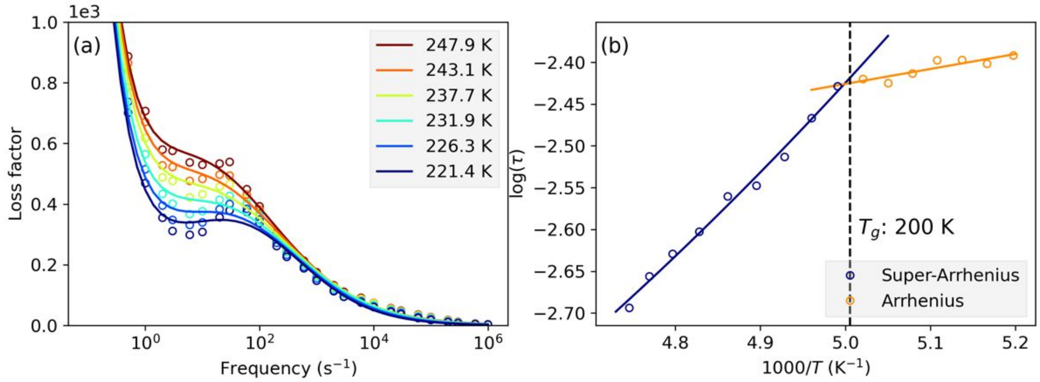

Figure S8 (a) IEPOX/2-MTS mixture (with 75wt% of IEPOX) Dielectric Spectra and (b) relaxation time-temperature relation

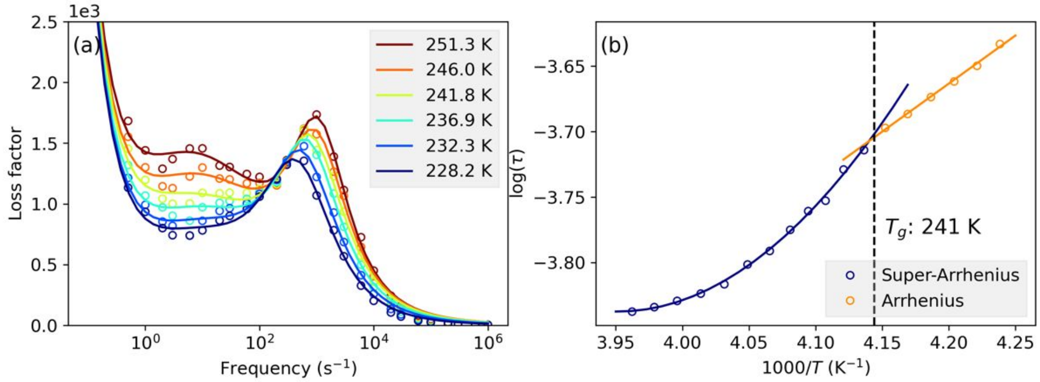

Figure S9 (a) IEPOX/3-MTS mixture (with 22.5wt% of IEPOX) Dielectric Spectra and (b) relaxation time-temperature relation

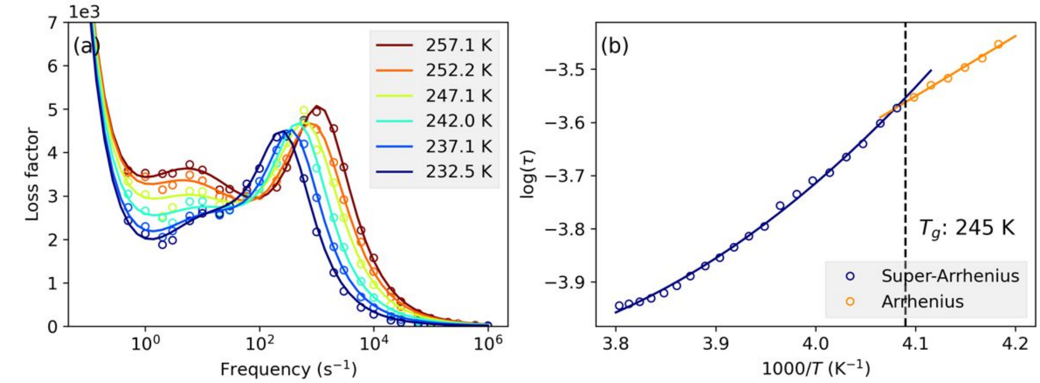

Figure S10 (a) IEPOX/3-MTS mixture (with 40.2wt% of IEPOX) Dielectric Spectra and (b) relaxation time-temperature relation

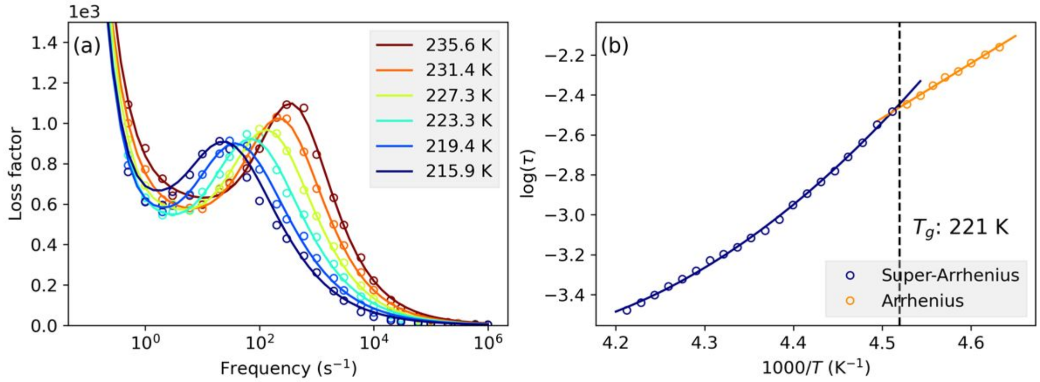

Figure S11 (a) IEPOX/3-MTS mixture (with 61.2wt% of IEPOX) Dielectric Spectra and (b) relaxation time-temperature relation

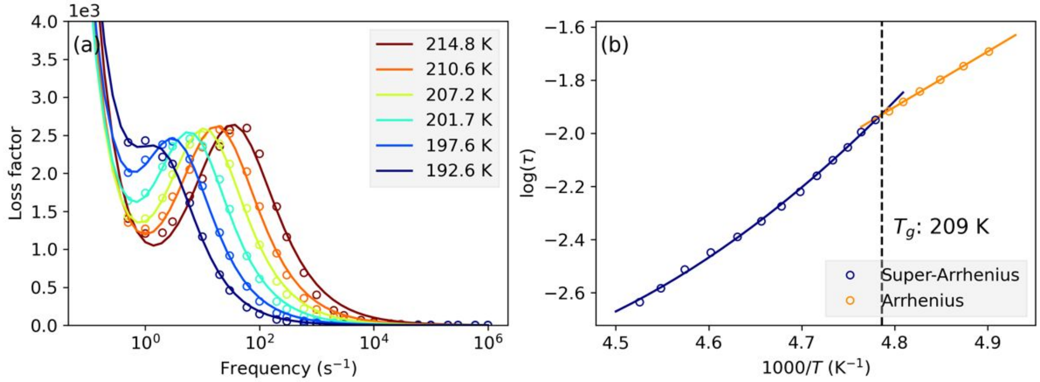

Figure S12 (a) IEPOX/3-MTS mixture (with 80wt% of IEPOX) Dielectric Spectra and (b) relaxation time-temperature relation

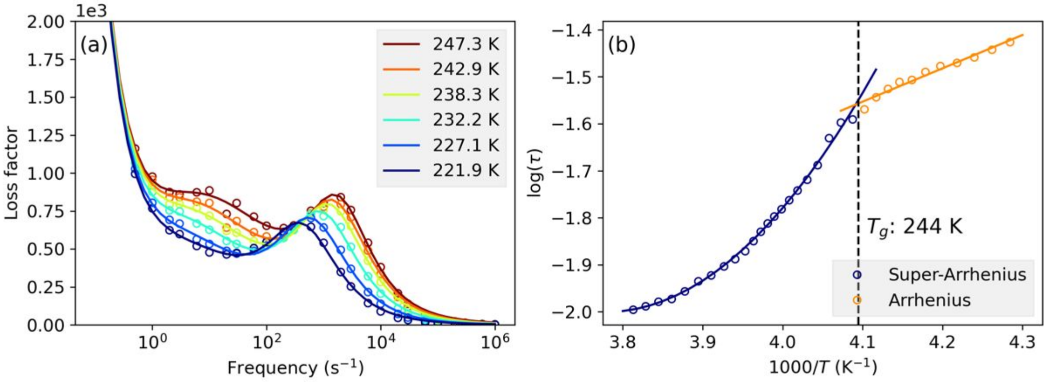

Figure S13 (a) 2-MT/2-MTS mixture (with 19.7wt% of 2-MT) Dielectric Spectra and (b) relaxation time-temperature relation

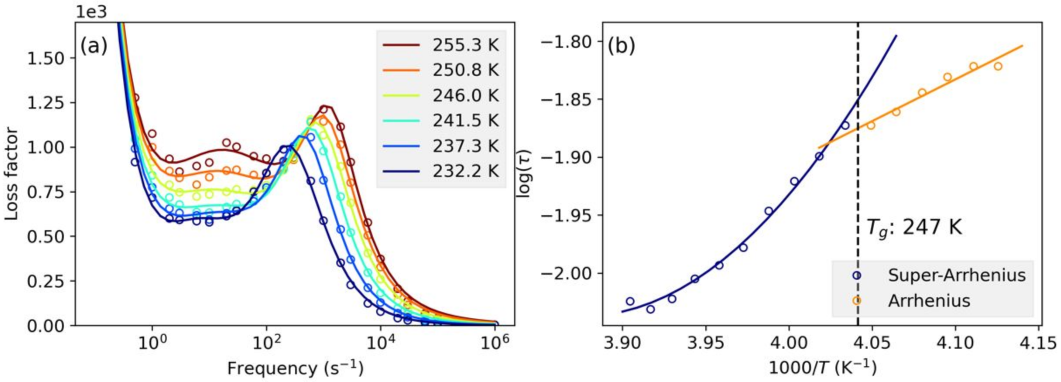

Figure S14 (a) 2-MT/2-MTS mixture (with 37.1wt% of 2-MT) Dielectric Spectra and (b) relaxation time-temperature relation

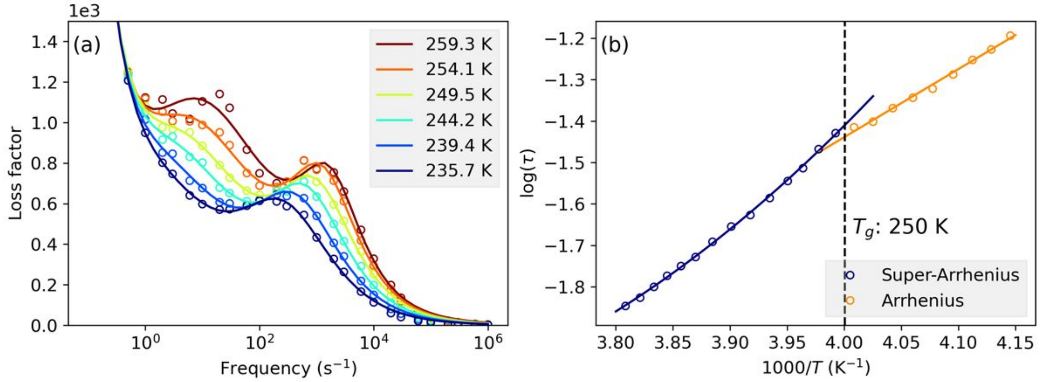

Figure S15 (a) 2-MT/2-MTS mixture (with 63.0wt% of 2-MT) Dielectric Spectra and (b) relaxation time-temperature relation

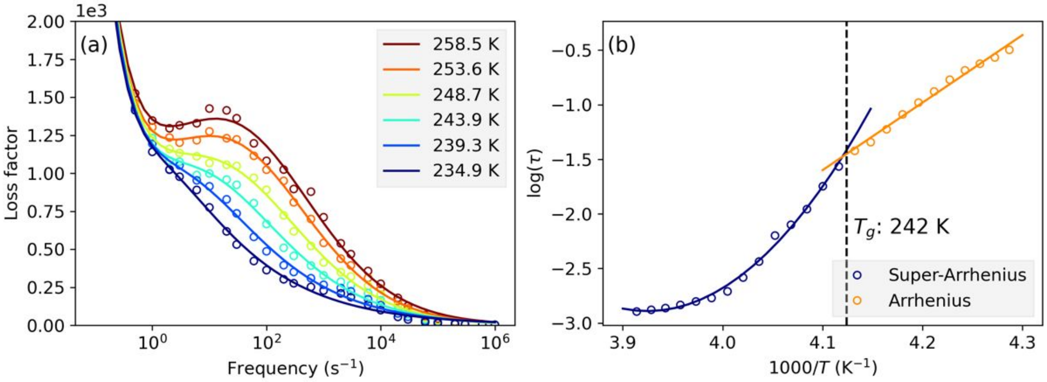

Figure S16 (a) 2-MT/2-MTS mixture (with 77.5wt% of 2-MT) Dielectric Spectra and (b) relaxation time-temperature relation

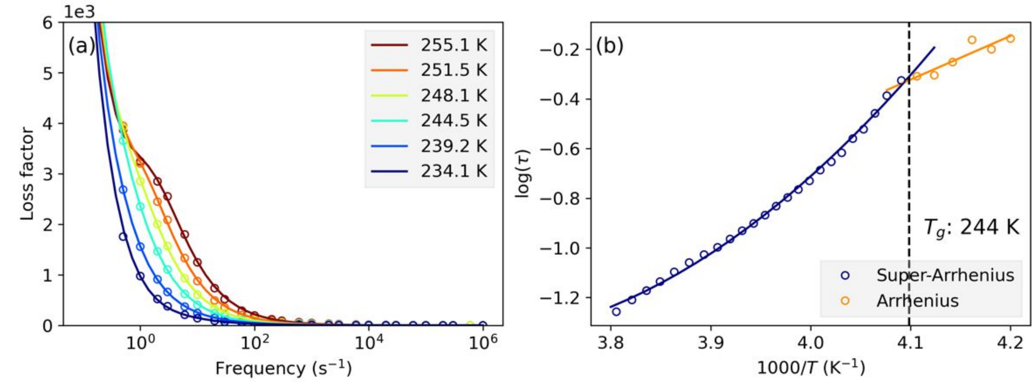

Figure S17 (a) 2-MT/3-MTS mixture (with 18.3wt% of 2-MT) Dielectric Spectra and (b) relaxation time-temperature relation

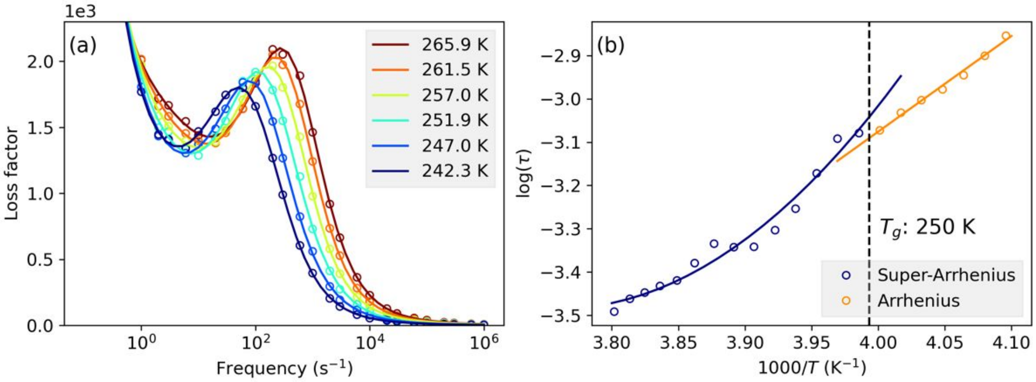

Figure S18 (a) 2-MT/3-MTS mixture (with 40.2wt% of 2-MT) Dielectric Spectra and (b) relaxation time-temperature relation

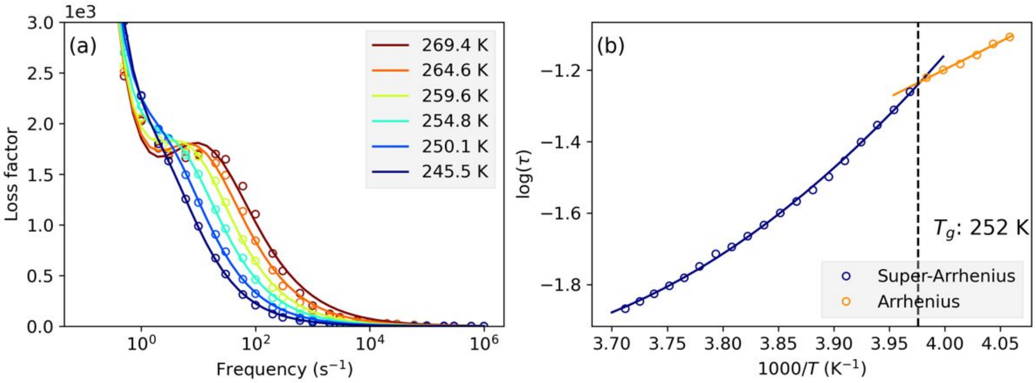

Figure S19 (a) 2-MT/3-MTS mixture (with 51.5wt% of 2-MT) Dielectric Spectra and (b) relaxation time-temperature relation

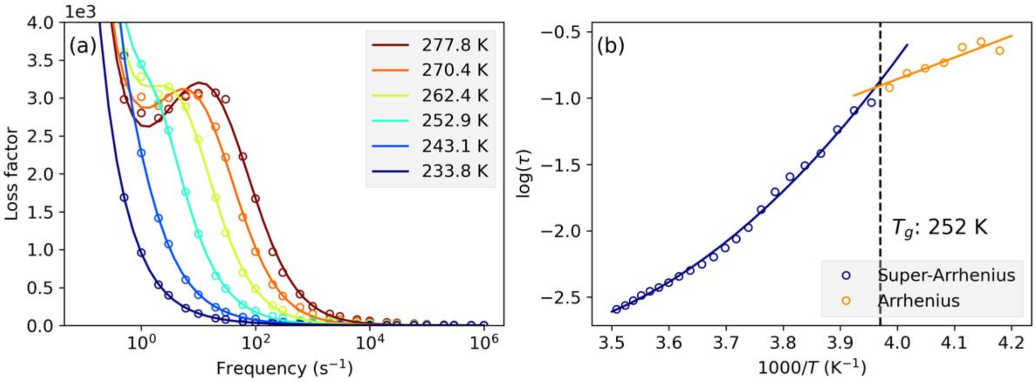

Figure S20 (a) 2-MT/3-MTS mixture (with 59.6wt% of 2-MT) Dielectric Spectra and (b) relaxation time-temperature relation

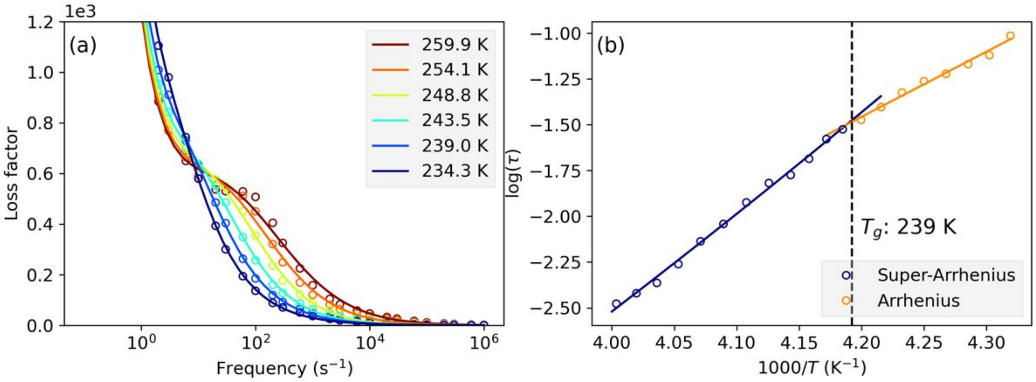

Figure S21 (a) 2-MT/3-MTS mixture (with 82.0wt% of 2-MT) Dielectric Spectra and (b) relaxation time-temperature relation

Supporting Figures for Raman Spectra Analysis

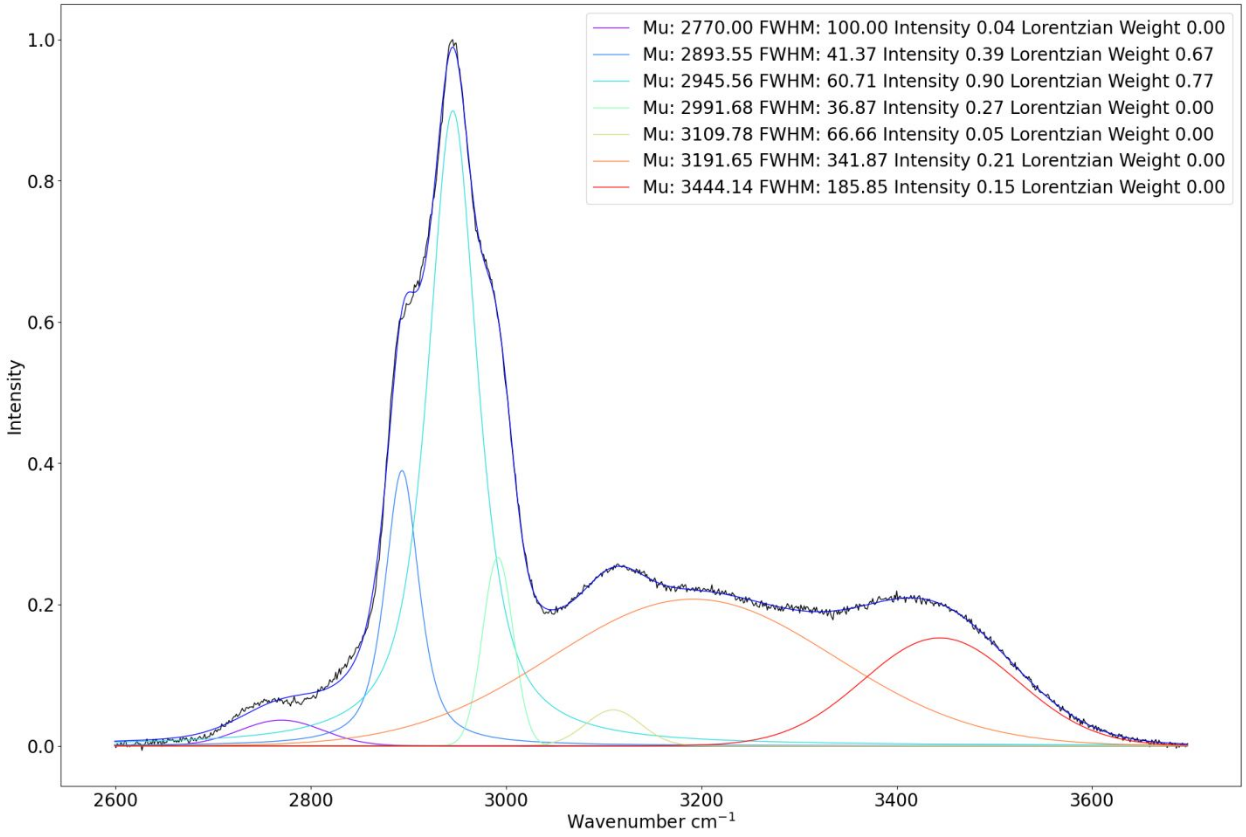

Figure S22 Decomposition of Raman spectra of 3-MTS from 2600 cm^{-1} to 3700 cm^{-1}.

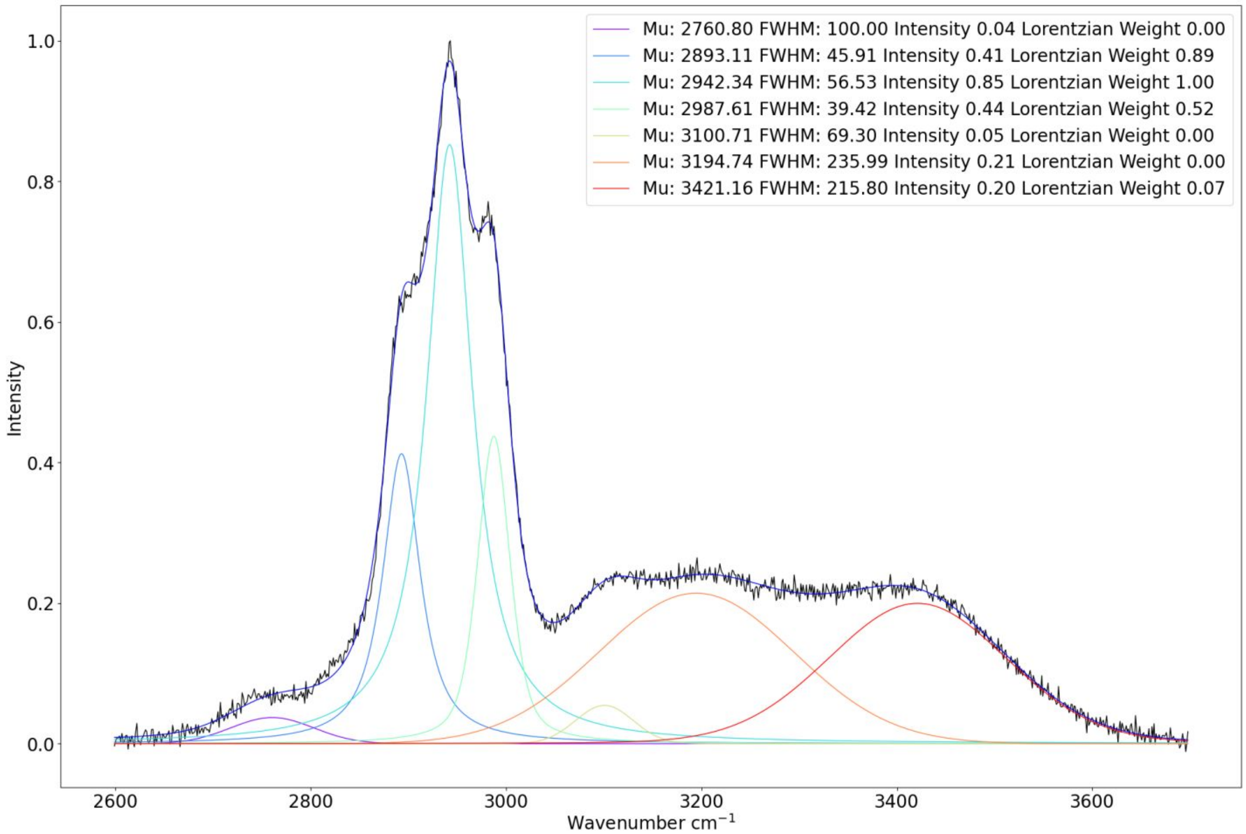

Figure S23 Decomposition of Raman spectra of 2-MT/3-MTS mixture (with 18.3wt% 2-MT) from 2600 cm^{-1} to 3700 cm^{-1}.

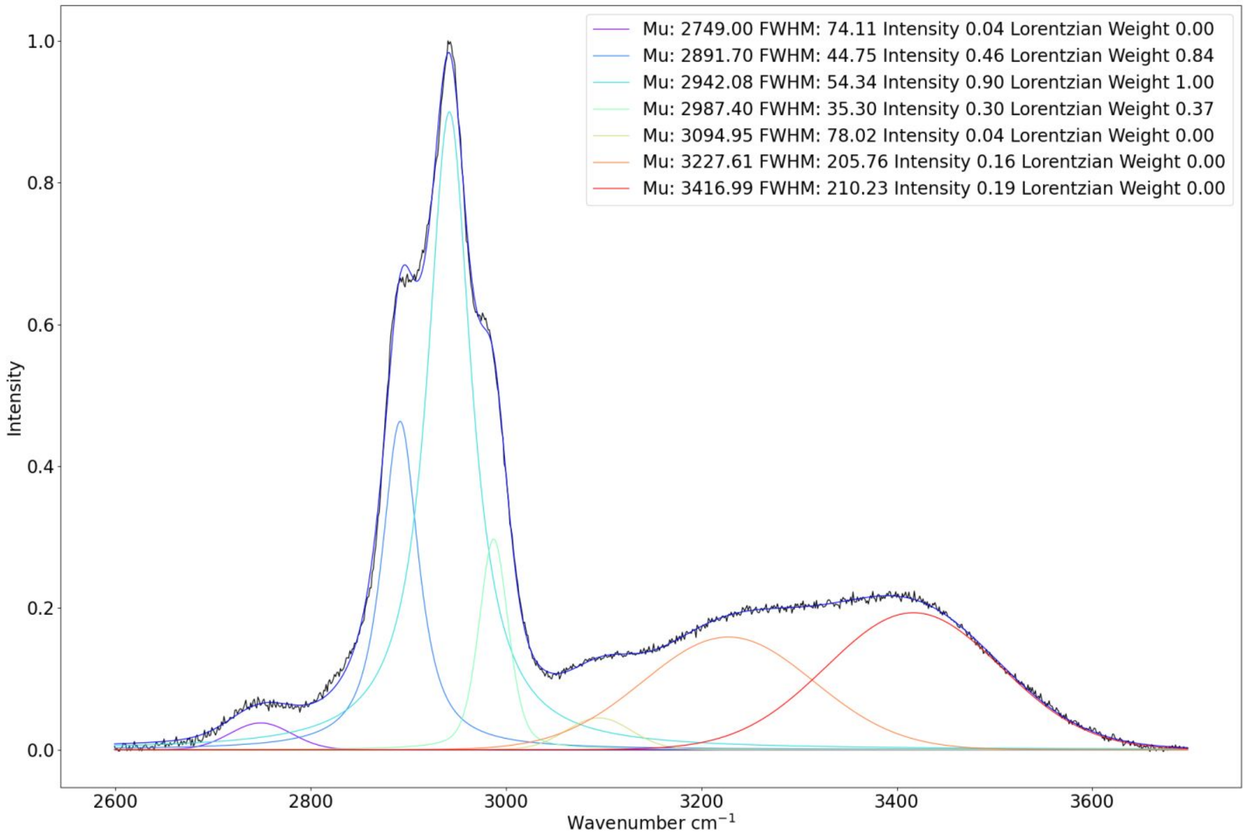

Figure S24 Decomposition of Raman spectra of 2-MT/3-MTS mixture (with 51.5wt% 2-MT) from 2600 cm^{-1} to 3700 cm^{-1}.

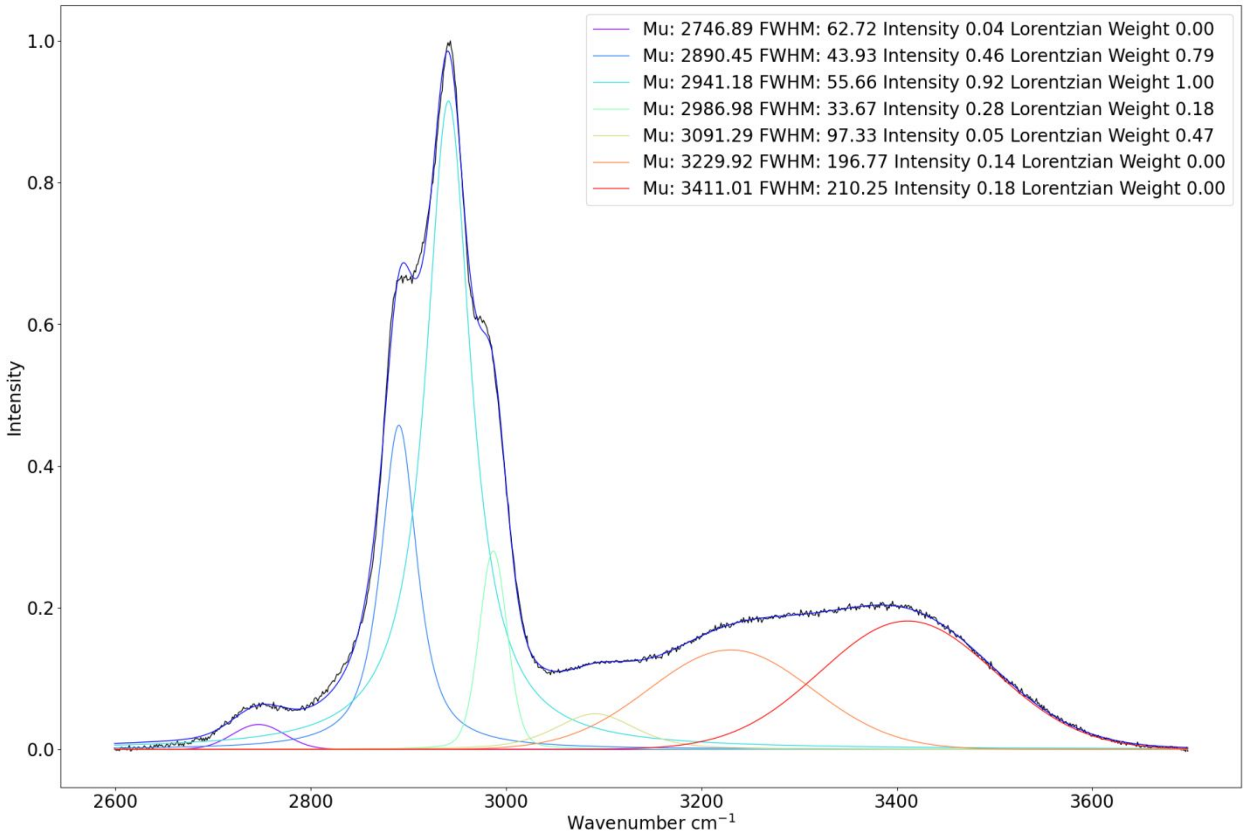

Figure S25 Decomposition of Raman spectra of 2-MT/3-MTS mixture (with 59.6wt% 2-MT) from 2600 cm^{-1} to 3700 cm^{-1}.

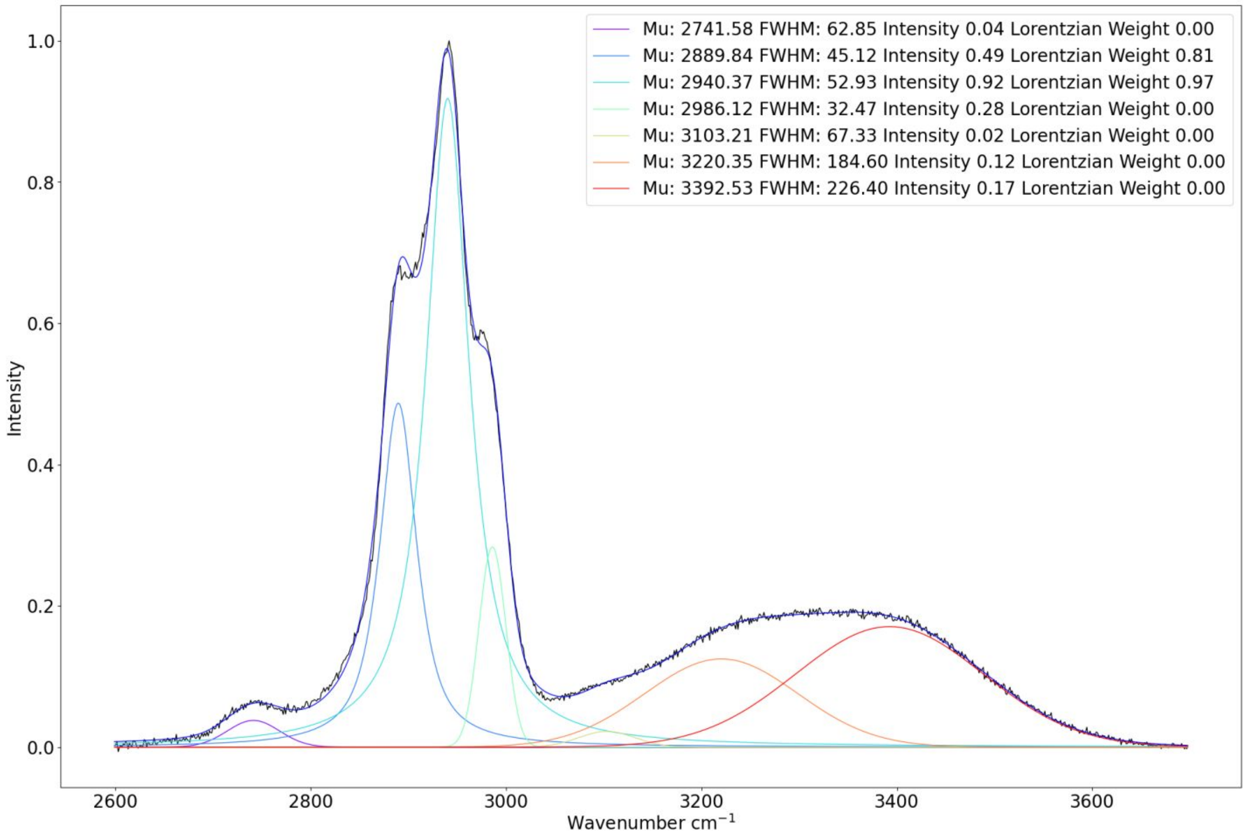

Figure S26 Decomposition of Raman spectra of 2-MT/3-MTS mixture (with 82.0wt% 2-MT) from 2600 cm^{-1} to 3700 cm^{-1}.

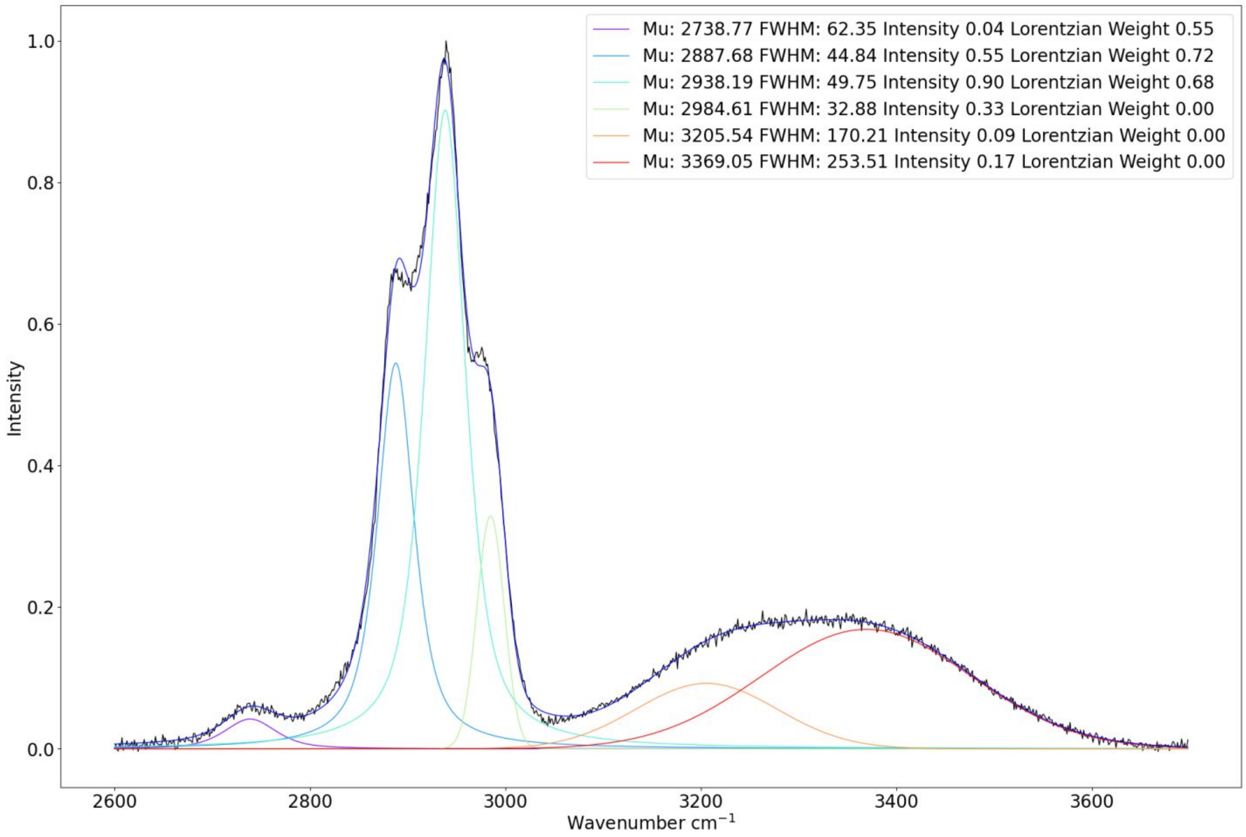

Figure S27 Decomposition of Raman spectra of 2-MT from 2600 cm^{-1} to 3700 cm^{-1}.
